# Burden of Middle‐Aged and Elderly Patients With Non‐Hodgkin Lymphoma From 1990 to 2021: A Systematic Analysis Based on the Global Burden of Disease 2021

**DOI:** 10.1002/cam4.71609

**Published:** 2026-03-11

**Authors:** Yuecan Chen, Yanjie Jiang, Yehan Xu, Yucao Ma, Wenjing Yao, Qinhan Cao, Xin Zhang, Liyuan Peng, Yaling Tang, Yuxin Cheng, Ruhua Ren, Xinyi Chen, Haiyan Lang

**Affiliations:** ^1^ Dongzhimen Hospital Beijing University of Chinese Medicine Beijing China; ^2^ Nanjing Hospital of Chinese Medicine Affiliated to Nanjing University of Chinese Medicine Nanjing China; ^3^ Hospital of Chengdu University of Traditional Chinese Medicine, Chengdu University of Traditional Chinese Medicine Chengdu China; ^4^ School of Basic Medical Sciences Lanzhou University Lanzhou China; ^5^ School of Basic Medicine Chengdu University of Traditional Chinese Medicine Chengdu China

**Keywords:** age‐standardized rate, estimated annual percentage change, global burden of disease, middle‐aged and elderly, non‐Hodgkin lymphoma

## Abstract

**Background:**

Non‐Hodgkin lymphoma (NHL) is increasingly prevalent in middle‐aged and elderly populations, contributing to the rising global health burden, particularly in high‐income countries.

**Methods:**

This retrospective analysis utilized data from the Global Burden of Disease (GBD) study (1990–2021) to examine temporal trends in age‐standardized incidence, mortality, and disability‐adjusted life year (DALY) rates of NHL among middle‐aged and older adult populations, incorporating age‐ and sex‐stratified comparisons. Furthermore, projections for the subsequent 30 years were generated using Bayesian age‐period‐cohort (BAPC) modeling.

**Results:**

From 1990 to 2021, the number of cases, deaths, and DALYs in middle‐aged and older people with NHL had significant increases. Age‐standardized incidence rates exhibited an increasing trend (estimated annual percentage change [EAPC] = 0.53), whereas mortality (EAPC = −0.33) and DALY rates (EAPC = −0.52) demonstrated a decreasing trend. The Western European region and high SDI countries are the core burden areas for middle‐aged and elderly populations with NHL globally. Lower‐middle SDI countries experienced the fastest increase in mortality (EAPC = 0.38) and DALY rates (EAPC = 0.22), while high‐SDI countries experienced the steepest decline in all metrics. The DALY burden showed its peak value at 65–69 years among middle‐aged and aged adults with NHL, while the morbidity and mortality burden reached their highest point at 70–74 years. When stratified by gender, the overall burden was higher in males than in females. High BMI causes the burden of NHL DALY to rise with increasing SDI, up to 39.14% in High‐income North America. By 2050, global NHL incidence is projected to increase modestly by 0.28%, whereas mortality and DALY burdens are expected to decline markedly by 30.67% and 23.02%, respectively.

**Conclusions:**

NHL in middle‐aged and older adults represents a significant global public health burden, necessitating context‐specific interventions due to variations across regions, genders, and age groups.

**Abbreviations**
ASDR: age‐standardized disability‐adjusted life year rate
ASIR: age‐standardized incidence rate
ASMR: age‐standardized mortality rate
ASR: age‐standardized rate
BAPC: Bayesian age‐period cohort
BMI: body mass index
CI: confidence interval
DALYs: disability‐adjusted life‐years
EAPC: estimated annual percentage change
GBD: global burden of disease
NHL: non‐Hodgkin Lymphoma
SDI: Sociodemographic Index
UI: uncertainty intervals

## 1 Introduction

Non‐Hodgkin lymphoma (NHL) is a diverse and complex group of hematologic malignancies that arise from lymphoid tissues, encompassing a wide range of histological subtypes, each with distinct clinical characteristics and prognoses. It accounts for about 90% of all malignant lymphomas, making it the most common type of lymphoma globally [1]. As reported in the Global Cancer Statistics 2022, NHL contributed to 553,010 new cases and 250,475 deaths worldwide, ranking it as the tenth most prevalent cancer globally [2]. This burden is expected to rise, with projections indicating that by 2040, the number of NHL cases could increase to 778,000 [3]. Although the overall survival rate for NHL has significantly improved due to recent advances in diagnosis and treatment [4, 5], the 5‐year survival rate for all forms of NHL remains at 72% [6]. The risk factors contributing to NHL are multifactorial and include genetic predispositions, environmental exposures such as pesticides, occupational hazards, immunosuppressive therapies, and viral infections like HIV [7, 8, 9]. These risk factors collectively underscore the complexity of NHL's epidemiology and highlight the need for ongoing research into its prevention, early detection, and treatment strategies.

However, it is essential to recognize that NHL is considered an age‐related condition, predominantly affecting middle‐aged and elderly individuals [10]. Globally, the highest proportion of NHL patients is observed in individuals over 50 years of age [11]. Across all age groups, the relative risk of NHL peaks at the age of 80 [12]. This trend assumes even greater significance in light of global demographic shifts: according to World Health Organization projections, the number of people aged 60 years and above will more than double—from 962 million in 2015 to an estimated 2.1 billion by 2050—while those aged 80 years and older are expected to nearly triple to 426 million [13]. The increasing aging population, with a significant rise in middle‐aged and older adults, will intensify the global burden of NHL [14]. Moreover, incidence and mortality rates of NHL among middle‐aged and elderly people vary by region and country and are greatly influenced by income level [12]. Given these converging factors—escalating elderly populations, uneven resource distribution, and the intrinsic vulnerability of aged immune systems—it is imperative to undertake comprehensive, age‐stratified analyses of NHL burden across diverse countries and regions, thereby guiding targeted prevention, screening, and treatment strategies for at‐risk older cohorts.

Previous studies of the global burden of NHL based on the GBD 2019 dataset have often failed to focus in depth on age‐specific trends in middle‐aged and older populations. Based on the most recent GBD 2021 data, this study systematically quantifies the global burden of NHL in middle‐aged and older adults from 1990 to 2020, examining the effects of SDI, geographic regions, age cohorts, sex, and key risk factors. The study provides policymakers and public health authorities with evidence‐based recommendations to optimize NHL prevention and management strategies for middle‐aged and older populations, drawing on a comprehensive epidemiological analysis. Collectively, these findings underscore the complexity of NHL epidemiology and highlight the necessity for continued research into its prevention, early detection, and therapeutic approaches.

## 2 Methods

### 2.1 Overview and Definitions

This study performed a retrospective analysis using information from the GBD 2021 dataset, which was accessed via an online search using the GHDx query engine (https://vizhub.healthdata.org/gbd‐results/). This dataset systematically assesses the disease burden across 204 countries and territories from 1990 to 2021, employing a consistent and comparable methodology that encompasses census, fertility, disease prevalence, mortality, and other health‐related data. GBD 2021 employs a Bayesian prior, regularization, and pruned meta‐regression technique (MR‐BRT) to correct for biases resulting from differences in case definitions and study methodologies across countries. The data were modeled using spatiotemporal Gaussian process regression methods, stratified by age, sex, year, and location. Detailed methodologies are outlined in the GBD 2021 capstone paper appendix [15, 16]. This study did not involve identifiable information on living individuals, nor did it involve any animal experiments, and all reports adhered to guidelines ensuring accuracy and transparency in health assessment reporting, providing data on disease incidence, mortality, and disability‐adjusted life‐years (DALYs).

In brief, data on NHL occurrence and fatalities in the middle‐aged and older adult population were retrieved from individual cancer registries and a composite cancer registry database. NHL is a fatal disease, so the incidence and mortality rates were considered to reflect the burden. The study was conducted on patients with NHL admitted to hospitals with ICD‐9/ICD‐10 codes for 9th and 10th revisions (200–200.9, 202–202.8, C82–C82.9, C83.0–C83.8, C84–C85.0, C85.2–C85.8, C86–C86.6, C96–C96.9, respectively) [17]. As the GBD database provides age intervals in consecutive 5‐year ranges from 5–9 to 90–94 years, the target population of this study was ultimately limited to only middle‐aged and older adults older than 55 years of age.

### 2.2 Measures of Disease Burden

GBD 2021 provides global, regional, and national incidence, mortality, and DALYs, both in terms of number of cases per 100,000 population and age‐standardized rates (ASR). ASRs are based on the GBD 2021 global standard population structure [18]. To estimate each indicator (e.g., incidence, mortality, or DALYs), 1000 values are generated. Intermediate results for each cause and region are aggregated at each computation step, and a bootstrapping method using replacement sampling is applied at each estimation step, facilitating the quantification of uncertainty in all epidemiological variables and their propagation. The 95% uncertainty intervals (UI) were computed by taking the 2.5th and 97.5th percentiles of estimated values [17]. DALYs were calculated by summing the years of life lost and years lived with disability. DALYs are important measurements of the cost of disease burden. A more comprehensive and comparable methodology for understanding the global burden of the NHL is available in the methods of the Appendix S1.

### 2.3 Statistical Analysis and Data Visualization

To conduct our analysis, we used the global data from the GBD 2021, which is a collection of 21 GBD regions and 204 different places or countries with sex, age, incidence, mortality and DALYs for all patients aged ≥ 55 years with NHL. Data were disaggregated by sex and age group, with the following age ranges: 55–59, 60–64, 65–69, 70–74, 75–79, 80–84, 85–89, 90–94, and 95+. Heterogeneity in quantifying the burden of NHL may arise from differences in the age structure across countries and regions. To mitigate the effect of demographic differences, age‐standardized incidence rates (ASIR), age‐standardized mortality rates (ASMR), and age‐standardized disability‐adjusted life‐year rates (ASDR) were used to assess the GBD and its trends for NHL in middle‐aged and older populations from 1990 to 2021.

The ASR for NHL in the middle‐aged and elderly population was calculated using the following equation:

$$\mathrm{ASR}=\frac{\sum_{i=1}^{A}\text{aiwi}}{\sum_{i=1}^{A}\mathrm{wi}}\times 100,000$$

𝑎_𝑖_: the age‐specific rate in the *i*th age group; *w*: the number of people in the corresponding *i*th age group among the standard population; *A*: the number of age groups [19].

Also, the trend over time for ASR was looked into with estimated annual percentage change (EAPC), this is Annualized Percentage Change. The regression model is first set as: Ln(ASR) = *α* + *βX* + *ε* (where *α* denotes the intercept, *X* represents the year, *β* indicates the linear trend of ASR, and *ε* is the error term) [20]. The EAPC and its 95% CI were then calculated using the formula: EAPC = 100 × [exp (*β*) − 1]. The logarithmic transformation of ASR enables the modeling of relative changes in rates over time, ensuring that trends follow a linear pattern. This method is commonly used in epidemiologic studies to estimate percent changes in incidence over time [21, 22]. If both the EAPC value and the lower limit of its 95% CI are > 0, increase in ASR is noted. Secondly, when the EAPC value and the upper bound of the 95% CI value are less than 0 at the same time, it means that the ASR is decreasing. If the 95% CI range contains 0, the ASR is said to be stable [23]. We also examined the sole risk factor associated with NHL mortality and DALYs recorded in the GBD database: high body mass index (BMI). Furthermore, a BAPC analytic model, shown to demonstrate high accuracy in disease trend prediction [24], was used to predict the future global burden of NHL from 2022 to 2050, including ASIR, ASMR, and ASDR. The SDI is a composite index that integrates the total fertility rate below 25, the average years of education for individuals aged 15 and above, and lags in per capita income distribution [25]. In this study, countries and geographic regions were grouped into five SDI categories (Table S1) to investigate the correlation between the burden of NHL and socioeconomic development within the middle‐aged and elderly populations [19]. All data analyses and result visualizations were performed using the open‐source software R version 4.4.1, with statistical significance determined at a *p*‐value threshold of less than 0.05. Information on the R package and detailed methods is provided in the Appendix S1. Further details on the methods outlined above can be found in the Appendix S1.

## 3 Results

### 3.1 Global NHL and Its Temporal Trends

Between 1990 and 2021, the number of new cases, number of deaths, and number of DALYs due to NHL in middle‐aged and older adults rose every year. In addition, the global ASIR [EAPC = 0.53 (95% CI: 0.35, 0.70)] (Table 1) for NHL demonstrates an increasing trend, whereas the ASMR [EAPC = −0.33 (95% CI: −0.45, −0.21)] (Table S2) and ASDR [EAPC = −0.52 (95% CI: −0.63, −0.41)] (Table S3) demonstrate statistically significant decreasing trends.

**TABLE 1:** Global and regional incidence of non‐Hodgkin lymphoma in middle‐aged and older populations between 1990 and 2021.

Location	Incident cases			Age‐standardized incidence rates		
	1990	2021	Change percent (%)	1990	2021	EAPC (95% CI)
Global	152,296.80 (144,773.45, 161,003.11)	425,428.70 (388,535.27, 455,601.18)	179.34	22.68 (21.56, 23.98)	28.63 (26.15, 30.66)	0.53 (0.35, 0.70)
Gender						
Male	83,351.18 (79,576.94, 90,138.32)	245,149.83 (223,632.76, 264,525.69)	194.12	26.76 (25.55, 28.94)	35.05 (31.97, 37.82)	0.75 (0.59, 0.90)
Female	68,945.63 (63,815.85, 72,685.45)	180,278.87 (159,862.50, 195,673.51)	161.48	19.15 (17.73, 20.19)	22.92 (20.33, 24.88)	0.22 (0.02, 0.43)
SDI						
High SDI	88,901.96 (84,346.03, 91,589.00)	204,615.31 (18,4218.71, 220,362.75)	130.16	47.68 (45.23, 49.12)	59.31 (53.39, 63.87)	0.32 (0.06, 0.59)
High‐middle SDI	30,141.57 (28,476.22, 32,311.75)	95,038.31 (85,099.67, 105,172.99)	215.31	17.47 (16.51, 18.73)	27.41 (24.55, 30.34)	1.41 (1.29, 1.53)
Middle SDI	18,887.78 (17,337.88, 22,238.42)	84,719.71 (74,895.42, 95,993.43)	348.54	10.88 (9.99, 12.81)	18.03 (15.94, 20.43)	1.62 (1.52, 1.72)
Low‐middle SDI	9387.71 (8116.57, 11,762.73)	29,724.80 (26,598.09, 37,327.50)	216.64	9.31 (8.05, 11.67)	12.33 (11.03, 15.48)	0.79 (0.73, 0.86)
Low SDI	4833.58 (3879.39, 5720.58)	10,961.12 (9520.15, 12,974.92)	126.77	12.96 (10.40, 15.33)	13.36 (11.60, 15.81)	−0.10 (−0.22, 0.02)
Regions						
Andean Latin America	906.82 (785.38, 1089.98)	8004.74 (6235.28, 10,219.33)	782.73	27.02 (23.40, 32.48)	80.80 (62.94, 103.16)	3.67 (3.48, 3.85)
Australasia	2275.22 (2119.81, 2430.59)	6616.09 (5698.98, 7587.72)	190.79	57.75 (53.81, 61.70)	74.89 (64.51, 85.89)	0.46 (0.14, 0.78)
Caribbean	1083.27 (1000.56, 1174.24)	2416.17 (2106.78, 2749.76)	123.04	25.14 (23.22, 27.25)	26.10 (22.76, 29.70)	0.30 (0.11, 0.48)
Central Asia	517.28 (477.67, 561.42)	1001.00 (875.45, 1131.15)	93.51	6.47 (5.97, 7.02)	6.88 (6.02, 7.77)	0.20 (−0.01, 0.42)
Central Europe	3896.36 (3711.37, 4117.34)	11,394.07 (10,382.01, 12,396.82)	192.43	14.69 (13.99, 15.53)	30.77 (28.04, 33.48)	2.37 (2.10, 2.65)
Central Latin America	1818.56 (1744.96, 1891.03)	9449.58 (8412.27, 10,501.56)	419.62	13.40 (12.86, 13.94)	22.10 (19.67, 24.56)	1.32 (1.19, 1.45)
Central Sub‐Saharan Africa	383.07 (276.36, 526.99)	972.53 (691.70, 1381.11)	153.88	10.19 (7.35, 14.01)	10.78 (7.67, 15.31)	0.06 (−0.09, 0.21)
East Asia	16,200.49 (13,978.50, 20,252.20)	78,295.20 (61,903.32, 95,646.65)	383.29	10.88 (9.38, 13.60)	19.97 (15.79, 24.39)	2.21 (1.98, 2.44)
Eastern Europe	6255.35 (6034.86, 6518.61)	17,212.65 (15,867.16, 18,623.73)	175.17	12.79 (12.34, 13.33)	27.73 (25.56, 30.00)	2.64 (2.27, 3.01)
Eastern Sub‐Saharan Africa	2485.93 (1996.79, 2952.33)	5535.65 (4638.52, 6760.94)	122.68	20.43 (16.41, 24.27)	20.47 (17.16, 25.01)	−0.22 (−0.33, −0.12)
High‐income Asia Pacific	8858.39 (8108.54, 9536.38)	36,813.70 (30,912.38, 42,384.02)	315.58	25.33 (23.19, 27.27)	52.22 (43.85, 60.12)	2.34 (2.15, 2.53)
High‐income North America	45,912.91 (43,113.03, 47,791.86)	82,273.08 (74,206.74, 87,785.45)	79.19	79.26 (74.43, 82.50)	73.11 (65.94, 78.01)	−0.93 (−1.28, −0.59)
North Africa and Middle East	3323.33 (2745.28, 4172.45)	13,485.16 (11,560.77, 16,505.97)	305.77	11.76 (9.71, 14.76)	17.69 (15.16, 21.65)	1.39 (1.33, 1.44)
Oceania	27.99 (21.98, 37.27)	78.14 (60.84, 103.83)	179.17	5.82 (4.57, 7.75)	6.33 (4.93, 8.41)	0.28 (0.24, 0.32)
South Asia	8827.29 (7357.70, 10,351.18)	30,039.10 (26,681.40, 36,037.62)	240.30	9.30 (7.75, 10.90)	12.10 (10.75, 14.51)	0.63 (0.49, 0.77)
Southeast Asia	4038.51 (3512.23, 5205.96)	14,121.85 (11,997.22, 19,197.11)	249.68	9.54 (8.30, 12.30)	12.33 (10.47, 16.76)	0.61 (0.51, 0.72)
Southern Latin America	1634.26 (1535.71, 1735.54)	3778.84 (3388.24, 4139.89)	131.23	20.63 (19.39, 21.91)	25.68 (23.02, 28.13)	0.57 (0.15, 1.00)
Southern Sub‐Saharan Africa	451.94 (377.17, 545.31)	1592.20 (1304.67, 1776.58)	252.30	10.21 (8.52, 12.32)	16.35 (13.40, 18.25)	1.58 (1.22, 1.96)
Tropical Latin America	2203.65 (2078.35, 2301.55)	8407.54 (7726.58, 8990.49)	281.53	14.55 (13.73, 15.20)	18.98 (17.44, 20.30)	0.60 (0.35, 0.86)
Western Europe	39,928.64 (37,734.79, 41,707.37)	90,515.39 (80,841.06, 99,095.36)	126.69	41.12 (38.86, 42.95)	60.69 (54.21, 66.45)	1.03 (0.75, 1.31)
Western Sub‐Saharan Africa	1267.52 (1018.60, 1491.00)	3426.03 (2629.78, 4002.71)	170.29	8.78 (7.06, 10.33)	10.66 (8.18, 12.45)	0.51 (0.43, 0.58)

*Note:* EAPCs represent the annual percentage change and their 95% confidence intervals in age‐standardized rates during 30 years from 1990 to 2021.

Abbreviations: EAPCs, estimated annual percentage changes; SDI, Sociodemographic index.

Specifically, the total number of new cases globally increased from 152,296 (95% UI: 144,773.45, 161,003.11) in 1990 to 425,428 (95% UI: 388,535.27, 455,601.18) in 2021, representing an increase of 179.34% (Table 1). The number of deaths increased from 95,493 cases (95% UI: 89,968.07, 103,284.14) in 1990 to 204,021 cases (95% UI: 185,987.81, 219,599.59) in 2021, representing an increase of 113.65% (Table S2). The number of DALYs increased from 2000 (95% UI: 1,990,621.56, 2,298,029.47) in 1990 to 4,248,298 (95% UI: 3,922,407.88, 4,597,406.78) in 2021, representing an increase of 101.39% (Table S3).

In 2021, the gender distribution of middle‐aged and elderly NHL patients globally indicated a higher prevalence in males than in females. Globally, there were 245,149 (95% UI: 223,632.76, 264,525.69) new cases in males and 180,278 (95% UI: 159,862.50, 195,673.51) new cases in females (Ratio male vs. female = 1.36). There were 113,981 (95% UI: 104,091.59, 126,448.28) male deaths and 90,040 (95% UI: 78,730.55, 97,389.80) female deaths (Ratio male vs. female = 1.27). The number of DALYs was 2,459,901 (95% UI: 2,265,888.86, 2,739,042.35) in males and 1,788,397 (95% UI: 1,603,066.83, 1,934,623.96) in females (Ratio male vs. female = 1.38). This implies that under the same conditions, if there are 100 new cases and 100 deaths in females, the corresponding figures for males would be 136 new cases and 127 deaths, respectively (Table 1, Tables S2 and S3).

### 3.2 NHL in SDI Regions

**FIGURE 1:**
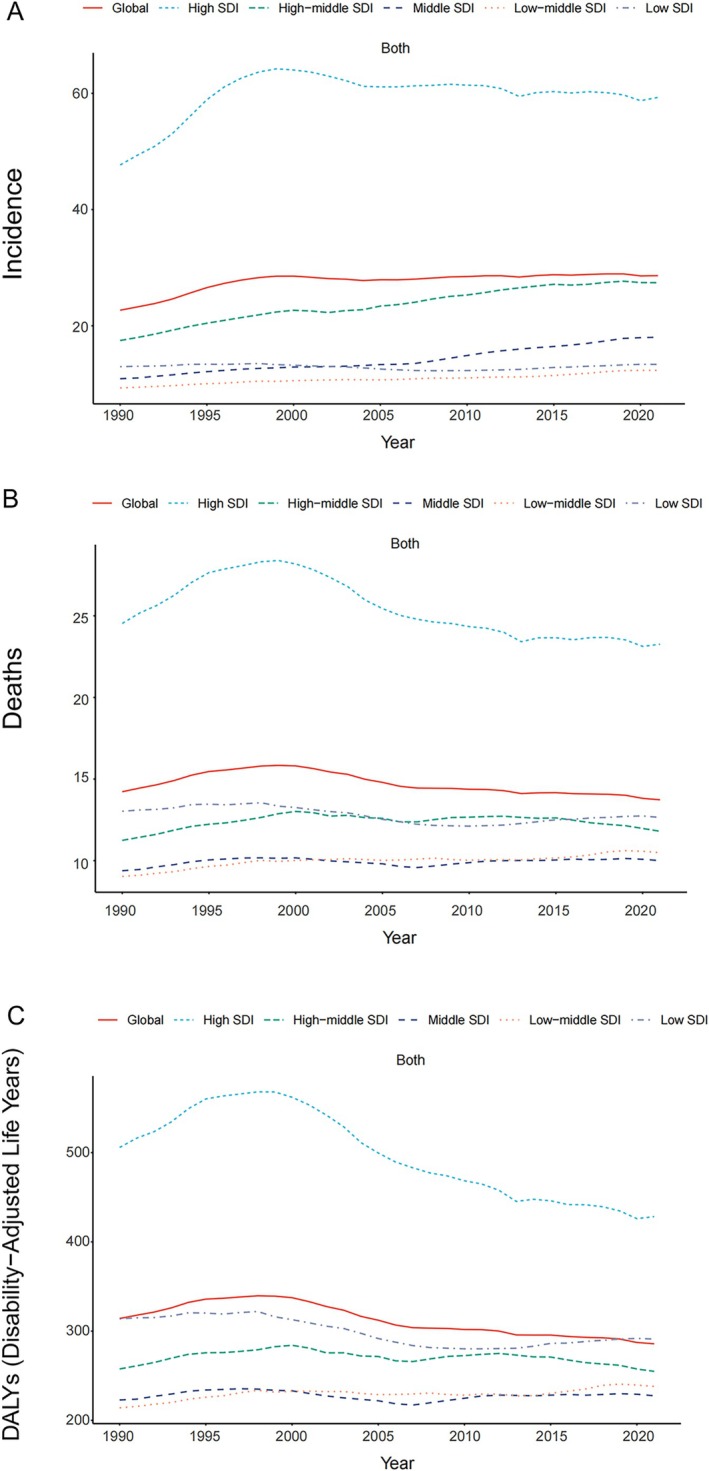
Epidemiological trends of age‐standardized (A) incidence, (B) mortality, and (C) DALY rates for middle‐aged and elderly NHL across 5 SDI regions from 1990 to 2021.

In 2021, the highest number of new NHL cases in middle‐aged and older adults was reported in the high‐middle SDI region (95,038 cases, 95% UI: 85,099.67, 105,172.99), while the lowest number occurred in the low SDI region (10,961 cases, 95% UI: 9520.15, 12,974.90). The highest increase in incidence compared to 1990 occurred in the middle SDI region, with a rise of 348.54%. Furthermore, the high SDI region exhibited the highest ASIR at 59 cases per 100,000 population. From 1990 to 2021, the ASIR in the five SDI regions generally exhibited an increasing trend, with the most significant rise in the middle SDI region [EAPC = 1.62 (95% CI: 1.52, 1.72)], and a declining trend in the low SDI region [EAPC = −0.10 (95% CI: −0.22, 0.02)], which was not statistically significant (Table 1, Figure 1A).

In terms of fatal cases, the highest number of deaths occurred in the high SDI region (80,252 deaths; 95% UI: 70,773, 85,684), while the lowest number was in the low SDI region (10,378 deaths; 95% UI: 9007, 12,332). The middle SDI region exhibited the largest increase in the number of deaths compared to 1990, with a rise of 188.55%. Additionally, the high SDI region exhibited the highest ASMR at 23 cases per 100,000 population. Between 1990 and 2021, three SDI regions showed an upward trend, with the low‐middle SDI region experiencing the largest increase [EAPC = 0.38 (95% CI: 0.30, 0.45)], while two regions showed a decreasing trend, with the high SDI region registering the largest decline [EAPC = −0.56 (95% CI: −0.74, −0.38)], both statistically significant (Table S2, Figure 1B).

In terms of DALYs, the high SDI region had the highest number of DALYs (1,477,836, 95% UI: 1,336,293.33, 1,568,490.91), while the low SDI region had the lowest number (238,845, 95% UI: 206,869.88, 285,210.25). The largest increase in DALYs occurred in the middle SDI region, with a rise of 176.56%. Furthermore, the high SDI region exhibited the highest ASDR of 428 cases per 100,000 people. Notably, from 1990 to 2021, ASDR increased only in the low‐middle SDI region [EAPC = 0.22 (95% CI: 0.14, 0.29)], while ASDR decreased in the remaining four SDI regions, with the most significant decrease in the high SDI region [EAPC = −0.93 (95% CI: −0.11, −0.74)], all of which were statistically significant (Table S3, Figure 1C).

Overall, the high SDI region had the highest number of deaths and DALYs and led in the ASIR, ASMR, and ASDR metrics, while the low SDI region exhibited relatively low values across all of these metrics. In addition, from 1990 to 2021, the ASMR and ASDR for the burden of NHL in middle‐aged and older adults increased most dramatically in low and middle SDI regions, while these metrics declined most dramatically in high SDI regions.

### 3.3 NHL in Different GBD Regions

**FIGURE 2:**
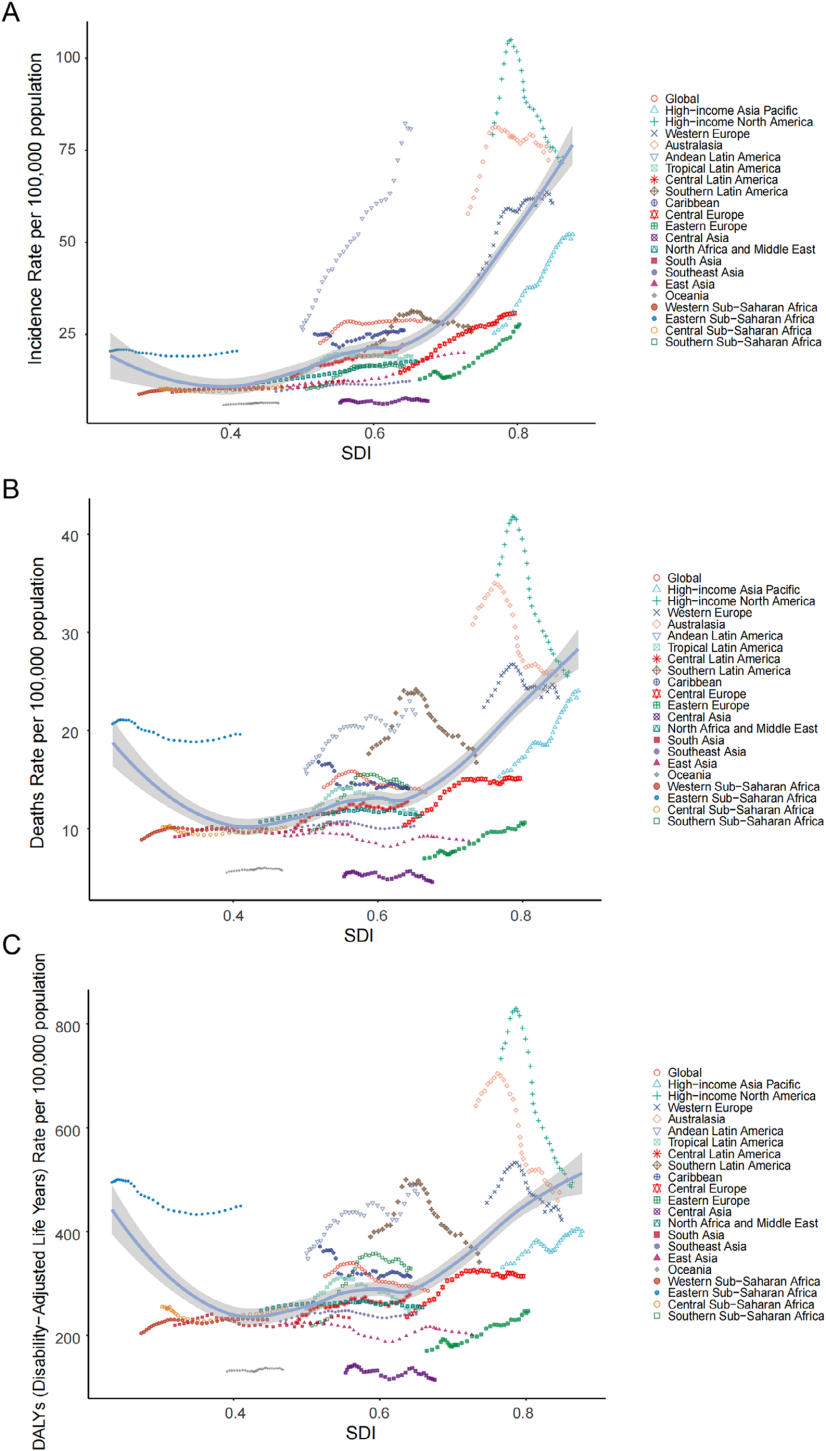
Age‐standardized rates of (A) incidence, (B) mortality, and (C) DALYs for middle‐aged and elderly individuals with NHL across 21 GBD regions, categorized by SDI, from 1990 to 2021.

In 2021, Western Europe had the most new cases of NHL among middle‐aged and elderly people, accounting for 20% of the global incidence in this age group (90,515/425,429 = 21.28%). It was followed by High‐income North America (82,273 cases) and East Asia (78,295 cases), together accounting for 60% of the cases around the world. The number of Oceania was the least, which was only 78 cases. In these regions, the ASIR was highest in the Andean Latin America at 80.80 cases per 100,000 people and the lowest in Oceania. Between 1990 and 2021, 17 GBD regions exhibited a significant upward trend in ASIR, with Andean Latin America [EAPC = 3.67 (95% CI: 3.48, 3.85)] and Oceania [EAPC = 3.67 (95% CI: 3.48, 3.85)] showing significant growth. Eastern Europe [EAPC = 2.64 (95% CI: 2.27, 3.01)] and Central Europe [EAPC = 2.37 (95% CI: 2.10, 2.65)] were also observed to show significant increases. Additionally, two GBD regions showed downtrends: Eastern Sub‐Saharan Africa and High‐income North America had an EAPC of −0.22 (95% CI: −0.33, −0.12) and −0.93 (95% CI: −1.28, −0.59), respectively (Table 1, Figure 2A).

The GBD region with the highest number of middle‐aged and elderly NHL deaths was Western Europe (34,874 cases), while Oceania had the fewest (71 cases). High‐income North America not only had the highest ASMR of 25.96 cases per 100,000 persons, but also witnessed the most precipitous ASMR drop, with a change score of [−1.69 (95% UI: −1.96, −1.42)]. Additionally, Central Asia showed the lowest ASMR of 4.58 deaths per 100,000 population. Between 1990 and 2021, there have been 10 GBD regions having had considerable growth in ASMR, with Eastern Europe showing the largest increase [EAPC = 1.45 (95% CI: 1.31, 1.60)]. Additionally, there were 7 GBD regions that experienced a drop in ASMR, with High‐income North America showing the most significant decrease [EAPC = −1.69 (95% CI: −1.96, −1.42)] (Table S2, Figure 2B).

The GBD region with the highest number of DALYs was taken by East Asia (791,285 cases), while Oceania had the lowest number (1653 cases). Additionally, High‐income North America had 494.38 cases per 100,000 thousand people, the highest ASDR, while Central Asia had the lowest at 128.10 cases per 100,000 people. Between 1990 and 2021, nine GBD regions witnessed considerable growth in ASDR; Southern Sub‐Saharan Africa reported the most substantial surge [EAPC = 1.42 (95% CI: 1.00–1.84)], and eight GBD regions were decreasing, among which High‐income North America saw the most significant drop [EAPC = −1.90 (95% CI: −2.15, −1.65)] (Table S3, Figure 2C).

Figure 2 illustrates the ASIR, ASMR, and ASDR of middle‐aged and older adults with NHL and their relationship with SDI across 21 GBD regions between 1990 and 2021. ASIR, ASMR, and ASDR exhibited a “U‐shaped” relationship with SDI. At low SDI levels, ASIR, ASMR, and ASDR were negatively correlated with SDI, reaching a minimum at SDI values between 0.40 and 0.45. Subsequently, as SDI levels increased, ASIR, ASMR, and ASDR were negatively correlated with SDI. Subsequently, as SDI levels rise, a positive correlation emerges, which is particularly significant in high‐SDI regions.

Furthermore, Western Europe led in the number of new cases and deaths, East Asia had the highest number of DALYs, while Oceania exhibited relatively low values across all these indicators. Among the 21 regions, Among the 21 regions, high‐income North America has the highest ASMR and ASDR, while Central Asia has the lowest in this regard. It is worth noting that the ASIR, ASMR, and ASDR in High‐income North America displayed a significant decline over time.

### 3.4 NHL in Different Countries and Regions

**FIGURE 3:**
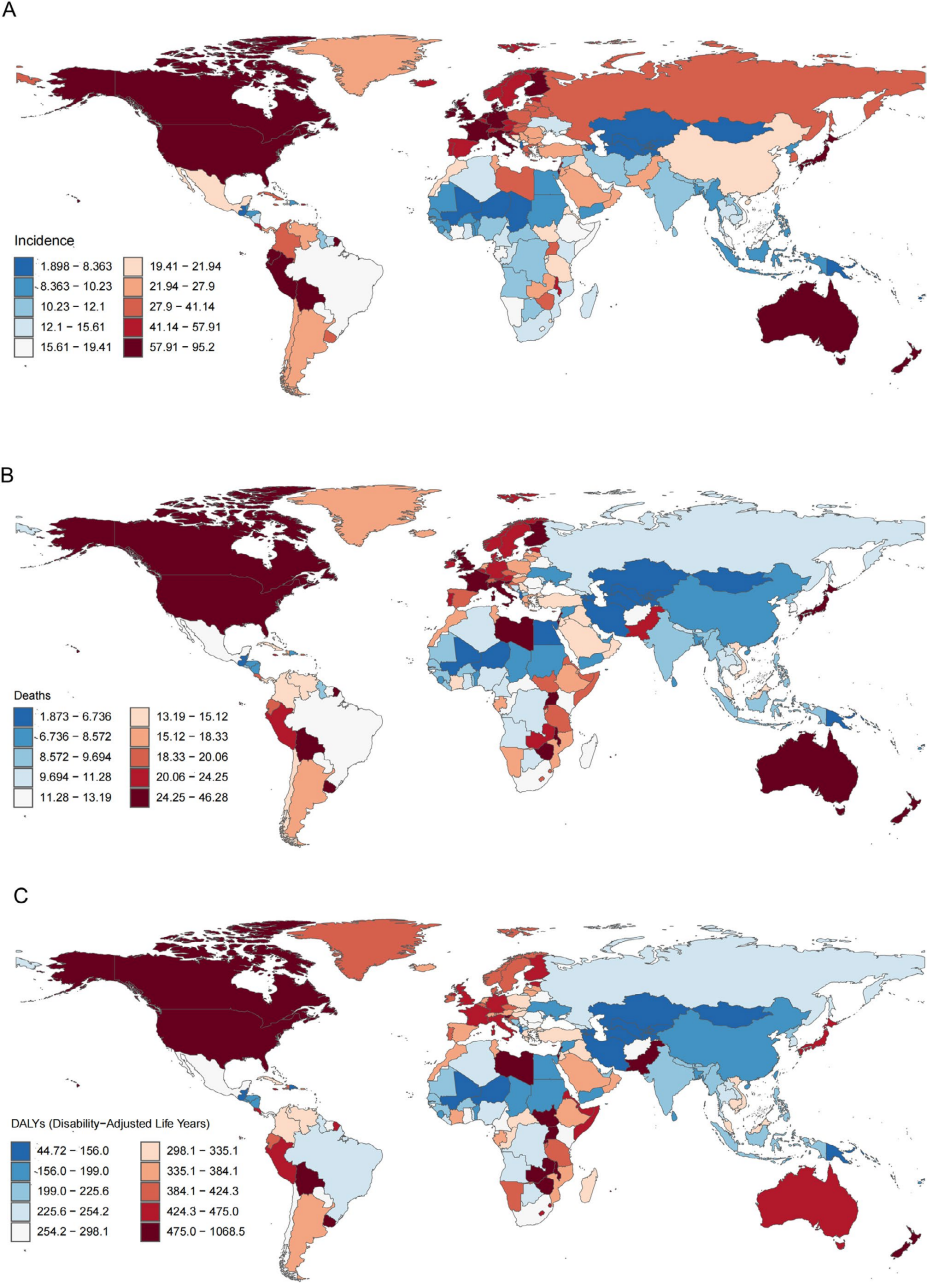
Age‐standardized rates of (A) incidence, (B) mortality, and (C) DALYs for middle‐aged and elderly individuals with NHL across 204 countries and territories from 1990 to 2021.

In 2021, China reported the highest number of new NHL cases among middle‐aged and older adults, accounting for nearly one‐fifth of the total global incidence (75,750/425,429 = 17.81%), followed by the United States (73,556) and Japan (31,488), together accounting for almost half (42.50%) of the global total. Conversely, Tokelau had the lowest incidence. Additionally, Peru had the highest ASIR, which was 95.20 cases per 100,000 people, followed by Slovenia (93.81) and New Zealand (86.25) per 100,000 people, while Kiribati had the lowest at 1.90 cases per 100,000 people (Figure 3A). Between 1990 and 2021, 151 countries or territories demonstrated a significant upward trend in ASIR, with Cabo Verde experiencing the greatest rise [EAPC = 5.53 (95% CI: 4.30, 6.77)], while 23 countries experienced a decline, with Burundi showing the greatest decrease [EAPC = −1.72 (95% CI: −1.96, −1.48)].

In terms of NHL deaths, China had the highest number of deaths at 32,482, and the United States had the next highest at 26,049, India the next highest at 19,461, and Tokelau the lowest. ASMR: Malawi had the highest, at 46.28 deaths/100,000, followed by Grenada (38.90) and Lebanon (32.58). In Kiribati, the lowest reported ASMR was 1.87 deaths per 100,000 people (Figure 3B). Between 1990 and 2021, 93 countries or territories exhibited a significant upward trend in ASMR, with Cabo Verde showing the greatest increase [EAPC = 4.84 (95% CI: 3.55, 6.15)]. On the contrary, 74 countries displayed a downward trend in ASMR, and Bahrain experienced the most substantial decrease [EAPC = −3.30 (95% CI: −3.79, −2.82)].

In terms of the burden of DALYs, China (753,978 cases), the USA (496,949 cases), and India (438,397 cases) were again in the top three, while Tokelau had the lowest (0.43, 95% UI: 0.32, 0.57). ASDR: In terms of ASDR, Malawi had the highest, having 1068.47 ASDR cases per 100,000. The following is Grenada with a rate of 896.83 cases for every 100,000 persons. Zimbabwe has the third highest ASDR with 748.67 cases. On the contrary, Kiribati had the smallest ASDR, which was 44.72 cases per 100,000 (Figure 3C). Between 1990 and 2021, 85 countries or territories exhibited a significant upward trend in ASDR, with Cabo Verde showing the greatest increase [EAPC = 4.80 (95% CI: 3.58, 6.04)]. Conversely, 78 countries experienced drops in ASDR, with Bahrain experiencing the most [EAPC = −3.17 (95% CI: −3.58, −2.75)].

Overall, China and the United States ranked in the top two in terms of new cases, deaths, and the number of DALYs, while Tokelau consistently ranked the lowest. Additionally, Malawi and Grenada ranked in the top two globally for both ASMR and ASDR. Notably, between 1990 and 2021, Cabo Verde exhibited a significant upward trend in ASIR, ASMR, and ASDR.

### 3.5 Age and Sex Differences in the Burden of NHL

**FIGURE 4:**
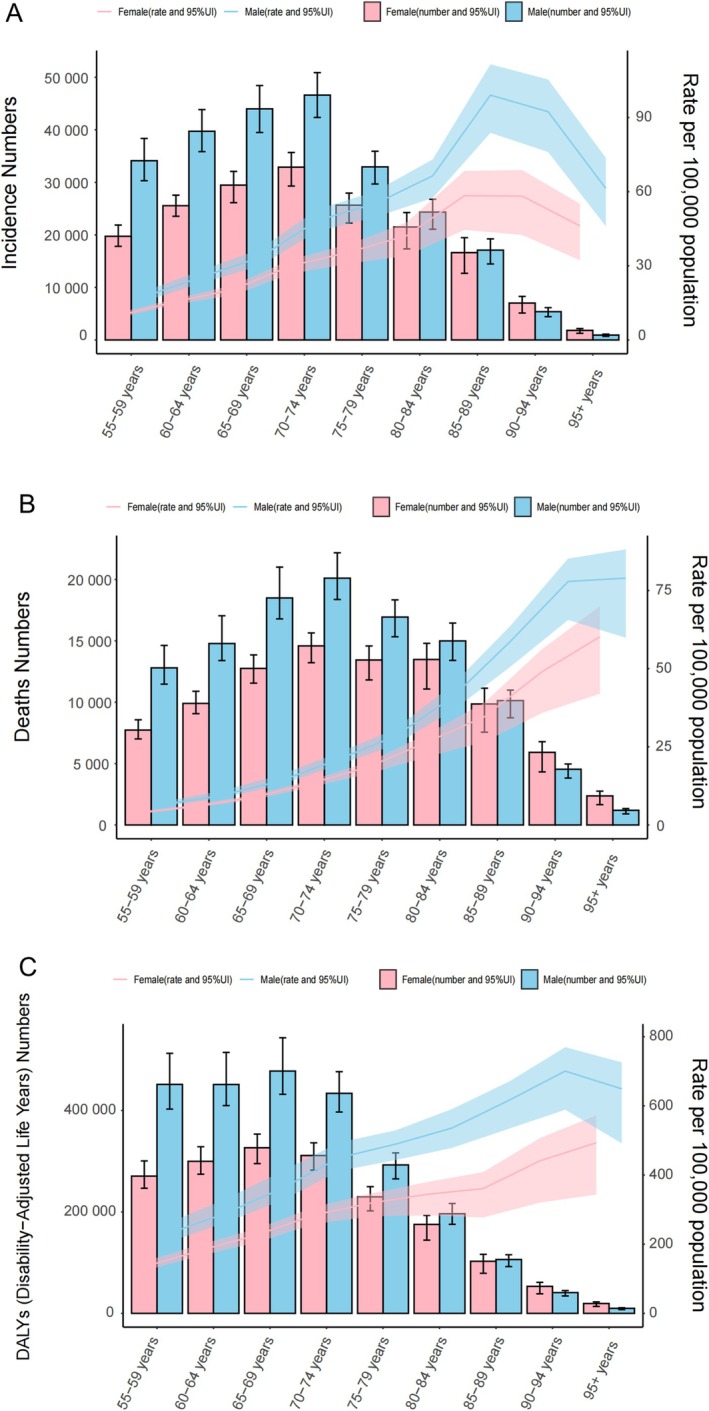
Counts and age‐standardized rates of (A) incidence, (B) mortality, and (C) DALYs for middle‐aged and elderly individuals with NHL in 2021, stratified by sex and gender.

In 2021, the number of newly diagnosed NHLs in middle‐aged people and older people was mainly concentrated in the group aged 70–74 years. In terms of age groups that are 55 and above, the ASIR for both men and women reaches its highest level in the 85–89 age group. Across all ages, the difference in ASIR was larger in males than in females, especially in the most extreme age group, that is, 85–89 with an ASIR difference of 40.70/100,000 people. From this age group onwards, the differences in ASIR diminished (Figure 4A). Death rates were highest in the 70–74 year‐olds for both sexes. As age increased, so did the ASMR, and the largest gender gap was in those aged 90–94 (Figure 4B). Additionally, the population of DALYs for middle‐aged and older adults with NHL peaked earlier, primarily concentrated in the 65–69 age group. ASDR in males continued to rise, peaking in the 90–94 age group before declining, while females consistently trended upward (Figure 4C). The overall burden of NHL was always greater in middle‐aged and elderly men compared with women under 90, while the reverse was true in the over‐90 age group. Notably, the 65–69 and 70–74 age groups are the primary age groups in which middle‐aged and elderly NHL DALYs, new cases, and deaths are concentrated. Therefore, the disease burden of middle‐aged and older NHL is primarily clustered in the 65–74 age range.

### 3.6 Epidemiological Risk Factors for NHL

**FIGURE 5:**
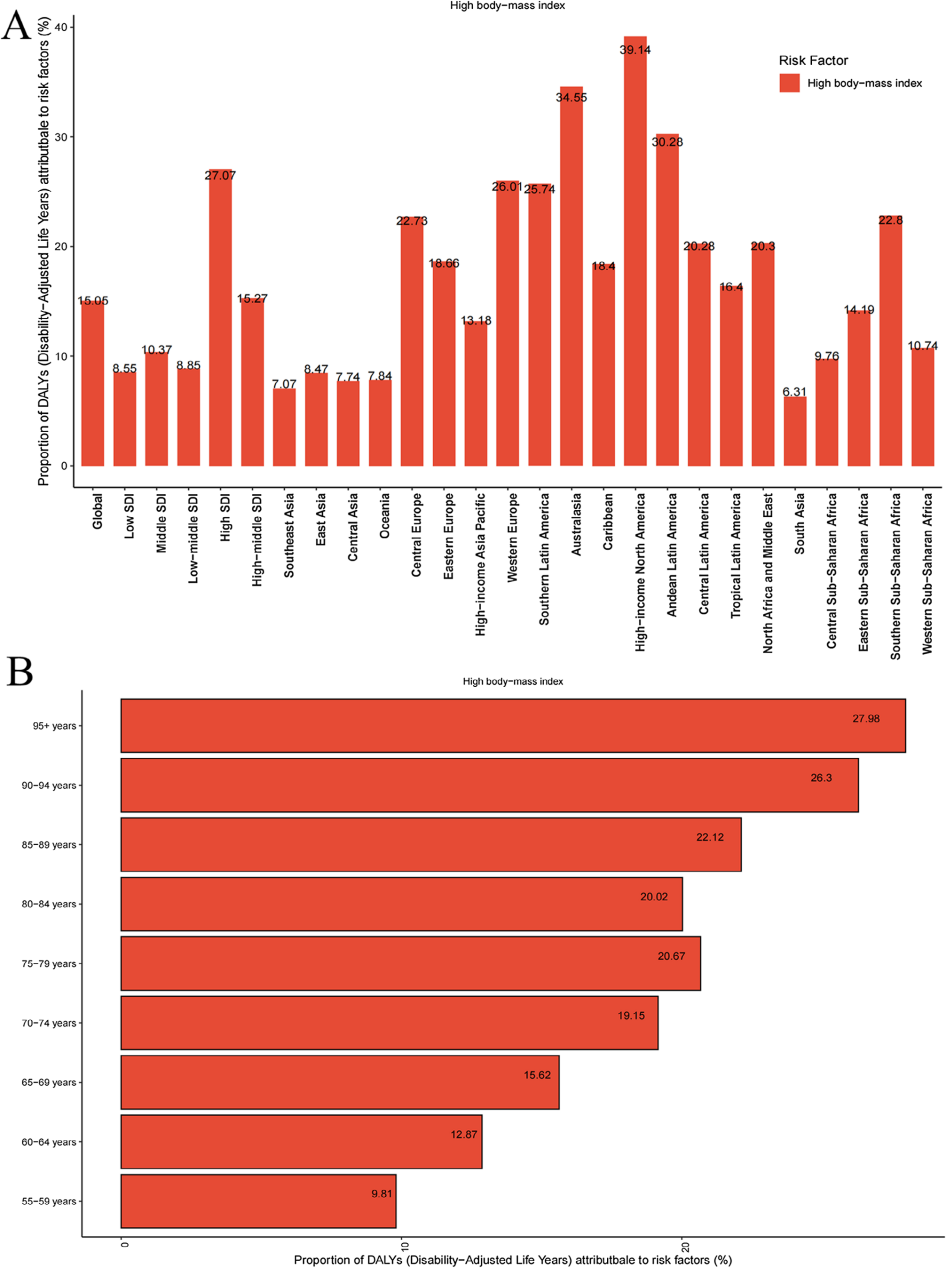
Proportion of DALYs among middle‐aged and elderly individuals with NHL attributable to elevated body mass index.

Notably, the GBD 2021 database lists high BMI as the only risk factor for NHL in middle‐aged and older populations. In 2021, the global burden of NHL in middle‐aged and older adults was significantly influenced by high BMI, which accounted for 15.05% of the DALY burden (Figure 5A). The share of DALYs linked to high BMI rose as SDI levels increased, reaching a maximum of 27.07% in regions with high SDI. There were significant regional variations in the proportion of the risk associated with high BMI, ranging from 6.31% to 39.14%. North America, with its higher income, had the largest share (39.14%). Additionally, the effect of high BMI on DALYs varied by age, with the 95+ age group experiencing the greatest impact, contributing to 27.98% of the DALY burden (Figure 5B).

### 3.7 Future Forecasts of Global Burden of NHL

**FIGURE 6:**
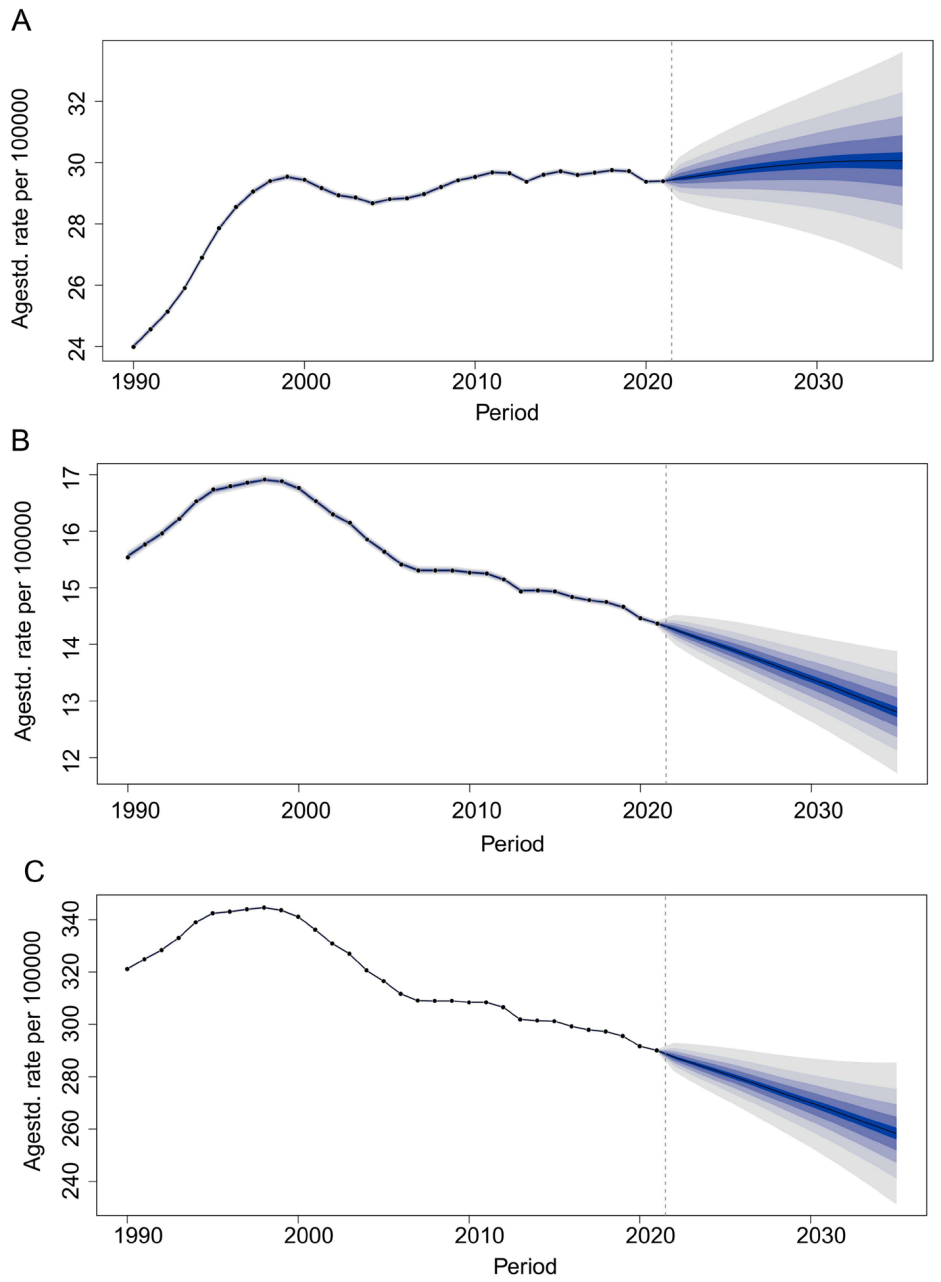
Projected global trends in age‐standardized (A) incidence, (B) mortality, and (C) DALYs rates of middle‐aged and elderly NHL from 2021 to 2050. The black line represents the global estimate, while the shaded area denotes the 95% UI, indicating its upper and lower bounds.

Based on the NHL burden data for middle‐aged and elderly populations from 1990 to 2021 in GBD 2021, the BAPC model was employed to forecast the temporal trends of ASIR, ASMR, and ASDR from 2021 to 2050. The projections suggest that the ASIR of NHL will continue to rise over the next three decades, whereas both ASMR and ASDR are expected to significantly decrease. From 2021 to 2050, the ASIR of NHL is projected to show a slow upward trend with a modest increase, reaching a peak of 30.06 per 100,000 people in 2035 before gradually declining, and then increasing by 0.28% in 2050 (Figure 6A). In contrast, the ASMR and ASDR of NHL in middle‐aged and elderly populations are projected to decrease significantly, reaching 10.99 and 223.26 cases per 100,000 people, respectively, a decrease of 30.67% and 23.02% compared to 2021 levels (Figure 6B,C). These projections suggest that with the significant decrease in ASMR and ASDR in middle‐aged and elderly populations, the global burden of NHL in these groups will continue to decline in the future.

## 4 Discussion

The GBD 2021 highlights the considerable worldwide load of NHL on persons in their middle years and later in life. Incidence, mortality and DALYs attributable to NHL in this demographic have increased markedly worldwide from 1990 to 2021. In 2021 alone, 420,000 middle‐aged and elderly people were diagnosed with NHL, resulting in 200,000 deaths, causing 4.24 million DALYs, but the effect of NHL on the population in different areas is different. Therefore, it is necessary to understand the regional differences in the NHL burden to effectively develop healthcare policies in different countries.

This study shows that there is a great deal of difference when looking at the number of people who get NHL in different places, and the countries that have the highest SDI have the biggest problem. In 2021, high SDI countries accounted for nearly half of the global incidence of cases, deaths, and DALYs among middle‐aged and older adults with NHL. In the 21 GBD regions, among the high SDI countries, western Europe and high‐income North America are consistently among the top in NHL‐related metrics for this demographic. This could be due to the aging of people in high SDI locations as well as improvements in diagnostic testing technology and greater prevalence of disease screening efforts. The increase in incidence may reflect better early detection of subclinical lesions [26, 27]. A Swedish study found that the five‐year prevalence of NHL increased by 47% from 2000 to 2016. The study attributed this increase, in part, to improved testing methods [28]. A 30‐year study (1984–2013) from Canada on incidence, survival, and mortality among different types of lymphoma found an increase in the incidence of NHL at a rate of 2.3% per year for men and 2.0% per year for women. The study suggests this may be due to an aging population, prevalence of risk factors, improved screening/diagnosis, or better cancer registries [29]. Additionally, the introduction of immunophenotypic and molecular criteria within the Revised European‐American, American Lymphoma, and the WHO classes could also have led to the recognition of previously unknown NHL subtypes, resulting in an overestimation of the occurrence [30, 31]. Furthermore, high‐income areas tend to report higher numbers of HIV infections [32]. HIV infection is a major risk factor for NHL [33]. Based on data from the Cancer and AIDS Registries of the United States, Italy, and Australia, the relative risk of NHL in HIV patients, compared to the general population, varies from 15 for low‐grade NHL to 400 for high‐grade NHL [34]. HIV infection further elevates the incidence of NHL. Specific NHL subtypes, such as Burkitt's lymphoma, are more prevalent in individuals with HIV and those who have undergone solid organ transplantation [35]. Additionally, common age‐related conditions like cardiovascular disease, diabetes, and cancer often coexist, potentially leading to higher mortality rates. These factors complicate treatment, influence therapeutic approaches, and place considerable strain on healthcare systems, particularly in high‐income regions [16].

Notably, over the past 30 years, despite the higher NHL disease burdens in high SDI regions like High‐income North America, there has been a significant downward trend in ASIR, ASMR, and ASDR for middle‐aged and older adults in this region. This trend predicts a gradual decline in the burden of NHL in middle‐aged and older adults in the future. This positive shift can be attributed to improved standards of care and therapeutic advances in high SDI regions. For instance, hematopoietic stem cell transplantation, small‐molecule–targeted inhibitors (such as BTK, PI3K, and BCL‐2 inhibitors), chimeric antigen receptor T‐cell therapy (CAR‐T), and bispecific T‐cell engager (BiTE) therapies have demonstrated remarkable efficacy in patients with refractory or recurrent NHL [36, 37]. Notably, CAR‐T therapy has yielded high complete remission rates and durable responses in relapsed/refractory B‐cell NHL, whereas bispecific T‐cell engager (BiTE) molecules have also exhibited significant antitumor activity in clinical trials [38, 39, 40]. Furthermore, the relatively lower incidence of NHL among middle‐aged and older adults in low‐income settings does not necessarily reflect a true decrease in new cases. In such environments, the burden of the disease may be underreported due to factors such as limited awareness, insufficient medical facilities, lack of proper disease screening, and incomplete registries [41]. This underreporting obscures the actual global burden of NHL and impedes equitable allocation of resources. Consequently, continued efforts are required to strengthen surveillance systems, expand oncology training, and develop cost‐effective diagnostic and therapeutic strategies adaptable to resource‐limited environments. International collaborations, capacity‐building initiatives, and telemedicine platforms may help bridge gaps, ensuring that emerging advances are not confined to high SDI contexts.

The heterogeneity in the incidence and trends of NHL among middle‐aged and older adults is also linked to the prevalence and distribution of known and presumed risk factors, including smoking [42], chemical exposure [43], infections [44, 45], and lifestyle factors [46]. Moreover, immunosuppressive therapy is a well‐established risk factor in middle‐aged and older adults. Prolonged administration of immunosuppressive agents impairs immune surveillance by reducing the detection and clearance of malignant cells. Consequently, the risk of lymphoid malignancies—including NHL—in this population is markedly elevated [47]. Patients with autoimmune diseases (e.g., rheumatoid arthritis) and organ transplant recipients often receive long‐term immunosuppressive therapy (e.g., methotrexate, cyclosporine, tacrolimus), which suppresses T‐cell function and elevates lymphoma risk [48, 49]. It has been shown that the relative risk of NHL in patients with rheumatoid arthritis can be increased by about 2–3 times [50, 51]; the incidence of NHL in organ transplant recipients is more than 10 times that of the general population [52]. High‐SDI regions may exhibit higher NHL incidence in middle‐aged and older adults owing to greater diagnostic capacity, higher prevalence of autoimmune disorders and transplantation, and more extensive immunosuppressive use; in contrast, low‐SDI regions show limited treatment coverage and a comparatively smaller impact on NHL burden. Notably, the disease burden of RA in high‐SDI regions parallels that of NHL [53, 54]. The absence of standardized, cross‐national data on immunosuppressant users in GBD 2021 constituted a key limitation precluding their incorporation into the quantitative model. Future studies should utilize national immunological disease and transplant registries to assess associations between immunosuppressive treatment cohorts and NHL incidence across different SDI levels. In recent years, both the prevalence of obesity and the incidence of NHL have risen concurrently, suggesting a potential link between obesity and an increased risk of developing NHL [55]. This perspective is further substantiated by the current study. The disease burden attributable to obesity is particularly pronounced in high‐income regions. One study forecasts that by 2030, nearly half of American adults will be obese [56]. The observed increase in NHL incidence in this study underscores the need for enhanced disease prevention preparedness in light of the current context of elevated risk factors. Thus, efforts must be directed toward incorporating effective preventive interventions into existing healthcare programs, as unhealthy lifestyles play a significant role in the development of chronic diseases and cancer. In summary, although the declining burden of NHL in regions with high SDI reflects the effectiveness of medical and systemic advancements, a comprehensive global strategy—incorporating enhanced surveillance, equitable access to diagnostics and therapies, and context‐specific research—is essential to accurately assess and ultimately mitigate the true worldwide impact of NHL among middle‐aged and older adults.

Age plays a crucial role in determining the burden of NHL in middle‐aged and older adults, influencing both the incidence and the severity of the disease. In the age‐stratified analysis of this study, individuals aged 70–74 years were found to be at the highest risk for both incidence and mortality associated with NHL. Meanwhile, those in the 65–69 age group experienced the most significant decline in quality of life due to the disease. This pattern reflects the heightened vulnerability of older adults to the effects of NHL, which is compounded by the fact that older patients typically have higher rates of comorbidities, malnutrition, frailty, and polypharmacy. These additional health issues complicate the decision‐making process regarding treatment options, such as whether to pursue aggressive medical therapies or opt for surgical interventions [57, 58, 59, 60, 61, 62]. As people age, these complexities only increase, making it more challenging to balance the risks and benefits of various treatment approaches. A Canadian cohort study highlighted these challenges, revealing that older individuals with aggressive NHL had higher rates of intensive care unit admissions and in‐hospital mortality, suggesting that the healthcare system faces considerable pressure when dealing with severe cases of NHL in elderly patients [63]. In terms of gender differences, this study found that globally, middle‐aged and older men bear a higher burden of NHL compared to women. This gender gap was found to peak at a certain age before gradually narrowing, a finding consistent with previous research [64]. The higher incidence and disease burden among men can likely be attributed to a combination of biological, social, and economic factors. For instance, NHL may progress differently in women due to gender‐specific biological factors such as hormonal changes and differences in immune system functioning [65, 66]. These findings underscore the importance of stratified management and personalized treatment strategies based on both age and sex to ensure that middle‐aged and older adults receive the most appropriate care tailored to their specific needs and circumstances. Such an approach is essential for improving overall health outcomes and enhancing the quality of life for these individuals, ultimately contributing to better disease management and a more holistic approach to care.

This study has several limitations. Similar to other diseases in the GBD study, the precision of the NHL disease model for middle‐aged and older adults is largely influenced by the quality and volume of the input data. In brief, although disease incidence and mortality data are available through the WHO for many countries, the accuracy of death cause recording and the completeness of registries vary significantly. In particular, data quality is poorer in some less developed regions, such as the tropical regions of Africa and Latin America, which lack reliable systems for reporting disease mortality and have fewer population‐based cancer registries. As a result, data quality, comparability, accuracy, and levels of missing data may vary significantly during data collection and analysis, leading to biased estimates, despite the use of multiple statistical methods for adjustment in this study. Moreover, GBD 2021 provides limited data on NHL risk factors and does not encompass all disease etiologies, highlighting the need to include additional relevant risk factors in future iterations. Furthermore, this study did not provide an extensive analysis of aging populations. Considering the distinct characteristics of these groups, further investigation into the burden of NHL among middle‐aged and older adults in aging countries could offer important insights. Nevertheless, this research represents the first thorough evaluation of NHL trends in these age groups globally over the past three decades, establishing a foundation for future studies and improving the quality and comparability of global NHL data.

## 5 Conclusions

NHL in middle‐aged and older people is still a big problem around the world, particularly in countries with a high SDI, where these folks keep being hit hard, even if overall rates aren't going up anymore. Simultaneously, the incidence and mortality of NHL in middle‐aged and older adults in newly industrialized and developing countries are both increasing. Furthermore, the disease burden varies significantly across gender and age groups. Given the impact of factors such as population aging, there is an urgent need for public health policymakers to acknowledge these trends and implement targeted interventions to effectively reduce the NHL burden.

## Author Contributions

**Yuecan Chen:** writing – original draft, writing – review and editing, methodology, data curation, supervision, investigation, funding acquisition, resources, formal analysis, visualization, project administration, validation, software, conceptualization. **Yanjie Jiang:** methodology, writing – review and editing, data curation, supervision, investigation, resources, visualization, project administration, validation, formal analysis, software, conceptualization, writing – original draft. **Yehan Xu:** writing – review and editing, writing – original draft, data curation, funding acquisition, resources, visualization. **Yucao Ma:** investigation, formal analysis, methodology. **Wenjing Yao:** validation, methodology. **Qinhan Cao:** data curation, visualization, writing – original draft. **Xin Zhang:** data curation. **Liyuan Peng:** writing – original draft. **Yaling Tang:** writing – review and editing, validation. **Yuxin Cheng:** conceptualization, formal analysis, validation. **Ruhua Ren:** supervision, visualization. **Xinyi Chen:** software, resources, writing – review and editing, methodology, investigation, validation, project administration, supervision. **Haiyan Lang:** writing – review and editing, resources, methodology, funding acquisition, supervision, visualization, validation, project administration, formal analysis.

## Funding

This study was supported by the National Natural Science Foundation of China (8247153912).

## Ethics Statement

The authors have nothing to report.

## Consent

The authors have nothing to report.

## Conflicts of Interest

The authors declare no conflicts of interest.

## Supporting information


**Data S1:** Supporting Information.

## Data Availability

The data sets generated and/or analyzed during the current study are available in the GBD repository (https://vizhub.healthdata.org/gbd‐results/).
