# Supplementary material for: Burden of Middle‐Aged and Elderly Patients With Non‐Hodgkin Lymphoma From 1990 to 2021: A Systematic Analysis Based on the Global Burden of Disease 2021
Source: Cancer Med. 2026 Mar 11;15(3):e71609. doi: 10.1002/cam4.71609 (PMC12976805; doi:10.1002/cam4.71609)
Supplement: Supplementary file 1 — Data S1: Supporting Information. [file CAM4-15-e71609-s001.docx]

**Supplementary material**

# eMethod:

In GBD 2021, the data processing workflow is a crucial step to ensure the accuracy and consistency of global disease burden estimates. This process includes systematic adjustments to epidemiological data to correct for biases arising from differences in data sources, definitions, and measurement methods. These adjustments are implemented using complex statistical models such as MR-BRT and DisMod-MR 2.1, ensuring internal consistency in estimates of incidence, prevalence, years lived with disability (YLDs), years of life lost (YLLs), and disability-adjusted life years (DALYs) across different regions, ages, genders, and years. This process aims to minimize heterogeneity in the study results through standardization and correction steps.

1. Data extraction criteria

Data were retrieved for the following univariates categorizations of stroke: (1) cause: Parkinson's disease defined according to the International Classification of Diseases 9/10 (ICD-9/10) or customized classifications in some countries; (2) location: global, 21 GBD regions, 5 regions classified based on the quartiles of the Socio-Demographic Index (SDI) and 204 countries and territories; (3) age: since the GBD 2021 database provides age intervals in consecutive 5-year intervals from 5-9 to 90-94 years of age, the target population for this study ultimately included people between the ages of 20 and 49, all ages and age-standardized; (4) sex: both, male and female; (5) year: from 1990 to 2019. Input data sources and results are available for download from the Global Health Data Exchange (<https://vizhub.healthdata.org/gbd-results/>).

2. Socio-demographic index (SDI)

SDI was originally constructed for GBD 2015 by using the Human Development Index (HDI) methodology, wherein a 0 to 1 index value was determined for each of the original three covariate inputs (TFR in ages 15 to 49 years, EDU15+, and LDI per capita) by using the observed minima and maxima over the estimation period to set the scales. In response to feedback from collaborators and the evolution of the GBD, we have refined the indicator with each GBD cycle. Beginning in GBD 2017, along with our expanded estimation of age‐specific fertility, we replaced TFR with TFU25 as one of the three component indices. The TFU25 provides a better measure of women’s status in society because it focuses on ages at which childbearing disrupts the pursuit of education and entrance into the workforce. In addition, we observed that in highly developed countries, the TFU25 has tended to decline consistently over time despite rebounds in TFR driven by increasing fertility at older ages. Thus, for each covariate input, an index score of 0 represents the minimum level of each covariate input past which selected health outcomes can get no worse, and an indexscore of 1 represents the maximum level of each covariate input past which selected health outcomes cease to improve. As a composite, a ocation with an SDI of 0 would have a theoretical minimum level of sociodemographic development relevant to these health outcomes, and a location with an SDI of 1 (before multiplying by 100 for reporting) would have a theoretical maximum level of sociodemographic development relevant to these health outcomes.

We computed the index scores underlying SDI as follows:

$$I_{cly}=\max\left( \frac{C_{ly}-C_{low}}{C_{high}-C_{low}}, 0.05 \right)$$

Where: $I_{cly}$ is the index for covariate C, location l and year y and is equal to the difference between the value of this covariate in this location year and the lower limit of the covariate divided by the difference between the upper and lower limits for this covariate. For access to SDI information, please visit the public webpage at http://ghdx.healthdata.org/record/ihme-data/gbd-2019-socio-demographic-index-sdi-1950-2019

3. Data processing and heterogeneity control

In GBD 2021, adjustments were made to epidemiological data known to have biases, such as those using alternative case definitions or measurement methods. These adjustments were made using correction factors estimated by MR-BRT (Meta-regression—Bayesian, regularized, trimmed), a collection of statistical models including linear and nonlinear mixed-effects models. The input data included paired estimates of two case definitions or measurement methods for the same age, sex, region, and year. MR-BRT also controlled for heterogeneity through network meta-regression and performed sex-splitting for inputs not reported by sex, and age-sex splitting for data not reported by either. These processes ensured data standardization and consistency, reducing heterogeneity issues caused by varying data sources and definitions. Additionally, input data spanning more than 25 years were disaggregated into finer age-specific estimates using alternative age patterns estimated from other available data sources.

4. Epidemiological estimation and YLD calculation

The prevalence and incidence estimates for most diseases and injuries were derived using DisMod-MR 2.1 (Disease Modeling Meta-Regression Tool, version 2.1). This Bayesian disease modeling tool generates internally consistent prevalence, incidence, remission, and mortality estimates stratified by sex, region, year, and age group. For areas lacking original epidemiological data, DisMod-MR 2.1 utilizes data from higher hierarchical levels as prior information to estimate parameters for lower levels. For certain causes, the Space-Time Gaussian Process Regression (ST-GPR) model was used as an alternative estimation method. For nonfatal causes, prevalence and incidence were further divided into specific sequelae estimates based on their severity. Sequela categories for nonfatal causes can range from asymptomatic to severe, depending on the disease. For most nonfatal causes, the proportion of cases in each sequela category was calculated using Medical Expenditure Panel Survey (MEPS) analysis. Crude YLD rates were estimated by multiplying the sequela-specific prevalence by the corresponding disability weights.

5. Comorbidity adjustment

GBD 2021 adjusted YLDs for comorbidities to account for the coexistence of nonfatal causes in the population, allowing YLDs to be additive within the GBD 2021 cause hierarchy. The coexistence of comorbidities was estimated through simulations involving 20,000 hypothetical individuals for each age, sex, region, and year. Each simulated individual was assigned the probability of having each sequela based on its prevalence. Subsequently, a cumulative disability weight was assigned to each individual by multiplying the disability weights of all assigned sequelae, with appropriate adjustments made to the sequela-specific disability weights.

6. YLLs and DALYs estimation process

In GBD 2021, YLLs were calculated by multiplying the estimated number of deaths by the standard life expectancy at the age of death, stratified by age, sex, region, and year. To ensure accurate attribution of causes of death, GBD 2021 employed the principles of the 11th edition of the International Classification of Diseases (ICD-11), assigning each death to the underlying cause that initiated the chain of events leading to death. For deaths recorded with non-specific, unreliable, or intermediate cause codes, reallocation algorithms were applied to reassign these "garbage codes" to the most probable causes of death. These algorithms were derived from published studies, expert consultations, or regression-based adjustments using data from sources reporting multiple causes of death. The cause of death for most diseases and injuries is estimated using the Cause of Death Ensemble model (CODEm). CODEm employs a set of statistical models, systematically testing the predictive validity of different covariate combinations, then combining the results to estimate the number of deaths for specific causes by location, age, sex, and year. For a small number of causes with sparse data or significant changes in reporting practices, GBD 2021 adopted customized modeling strategies, including the use of prevalence, incidence, case fatality data, or data related to sub-causes to infer causes of death. Through this process, GBD 2021 achieved progress in controlling data heterogeneity and reducing uncertainty. To estimate DALYs in GBD 2021, specific cause mortality rates and YLDs were first estimated. DALYs for each year were then calculated by adding YLLs to YLDs. The uncertainty of YLLs was assumed to be independent of the uncertainty of YLDs. By summing the first set of YLLs and YLDs across 500 simulations, and repeating the process for subsequent simulations, the 95% uncertainty interval for DALYs was ultimately calculated. The estimation of DALYs covered every cause, location, age group, sex, and year, providing a comprehensive assessment of the global health burden.

7. Bayesian age-period-cohort models (BAPC)

APC models estimate the individuals’ age, birth cohort and the period in which the event occurred: *η_ij_* = log(*λ_ij_*) = *μ* + *α_i_* + *β_j_* + *γ_k_* with intercept *μ*, and age, period and cohort effects *α_i_*, *β_j_* and *γ_k_*. *i* (1 ≤ *i* ≤ *I*) denotes the age group at time point *j* (1 ≤  *j*  ≤  *j*), the cohort index *k* depends on the age and period index as well as on the age group and period interval width: *k* = *j* + *M* (*I* − *i* ). *M* encodes the width of age groups as compared to period intervals, e.g. for 5 yr age groups and yearly data, *M* is 5. The BAPC model is a Bayesian model added to the age-period-cohort analysis model that can solve the difficulty of parameter estimation due to the linear relationship between the 3 factors in the age-period-cohort model. Age, period, and/or cohort effects can be optimized with second-order random walk (RW2) construction models to more accurately predict future ASIR, ASMR and ASDR. Separate age, period and cohort effects are not identifiable due to the exact linear dependence of effects.

The BAPC model is expressed as

n_ij_ = log(λ_ij_) = μ + α_i_ + β_j_ + γ_k_

where λ_ij_ denotes the count of cases, μ denotes the intercept, and αi, βj, and γk signify the effect of age, period, and cohort, respectively.

Using the posterior correlations between all η_ij_ , i = 1, . . . , I, j = 1, . . . , J, and the posterior standard deviations of each η_ij_ we derive the corresponding posterior covariance matrix Cov(η | y) of dimension (I · J) × (I · J). To derive the covariance matrix of λ| y we apply the multivariate delta rule:


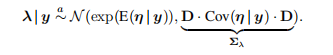


where the diagonal matrix D has exp(E(η | y)) on the diagonal. Having the posterior covariance matrix between age-specific mortality rates λ_ij_ , we are able to compute posterior standard deviations of λj as follows:


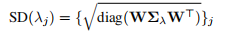


with WJ×(I·J) containing the age-specific weight wi at position Wj,(i−1)·I+j , j = 1, . . . , J. As summary statistics BAPC returns the age-standardized expected value


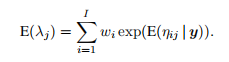


BAPC approximates a desired quantile to the given probability p using the corresponding quantile of a normal distribution with mean E(λ_j_ ) and variance SD(λ_j_ ). For age-standardisation we use the percentage of the population in each 5-year age group in the new WHO World Standard population as weights.

Using the law of iterated expectations the mean of the predictive distribution µ_ij_ can be derived. With yij | λ_ij_ ∼ Po(n_ij_ · λ_ij_ ) it follows:


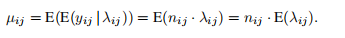


Analogously, the variance$\sigma_{\mathrm{ij}}^{2}$ = Var(y_ij_ ) follows from the law of total variance as


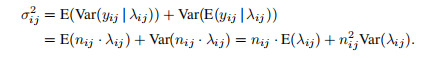


In the case of the age-standardized predictive distribution we obtain that


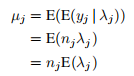


with nj = $\sum_{i=1}^{I} nij$. Analogously, the variance$\sigma_{\mathrm{ij}}^{2}$ = Var(y_ij_ ) follows from the law of total variance as


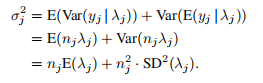


8. Hypothesis validation of the EAPC regression model

Regarding the necessary assumptions for applying the EAPC regression model, several key assumptions were verified to ensure the validity of the estimated trends. First, the assumption of linearity was tested by examining whether the relationship between the natural logarithm of ASR and year is indeed linear over the study period. Second, residual diagnostics were performed to assess the independence and homoscedasticity of the residuals, which are essential for the reliability of the regression model. Third, the presence of any significant autocorrelation in the residuals was tested to ensure the absence of temporal dependencies. Finally, the appropriateness of the model was further evaluated through sensitivity analyses, such as testing for non-linearity using polynomial regression or applying other trend estimation methods, which confirmed the robustness of the EAPC results. These verification steps were crucial to ensure that the assumptions of the regression model were satisfied and that the estimated trends accurately reflect the changes in ASR over time.

9. Processing of missing data

The processing of GBD 2021 data and the treatment of missing values follows a systematic approach, aiming to fill in data gaps across regions and time periods through a variety of statistical models. First, GBD aggregated data from population surveys, hospitalization registries, and national reporting systems to ensure breadth and representativeness. For missing raw data, GBD uses the DisMod-MR 2.1 model, which uses Bayesian regression to interpolate predictions by combining boundary data from neighboring regions and similar populations, as well as covariates that are closely related to the burden of disease (e.g., socio-demographic indices, life expectancy, and health expenditures as a percentage of GDP, etc.). The model not only outputs estimates of incidence and prevalence, but also provides their 95% uncertainty intervals at the same time to reflect potential errors and uncertainties. In addition, for the lack of mortality data, GBD used the CODEm (Cause of Death Ensemble model), which integrates multiple regression models such as linear regression, Poisson regression, and other regression models, and combines mortality satellite data with covariates such as socio-demographic indices, etc. The mortality rates and their uncertainty intervals output from the CODEm ensure the consistency of the estimation results for each region and time period. regions and time periods are comparable and adequately reflect differences in data quality. To further reduce fluctuations due to sparse data and underreporting, GBD also applies spatial-temporal Gaussian process regression (ST-GPR) for smoothing, thus optimizing the stability of the results. All outputs are subjected to 1,000 draws to generate uncertainty intervals, ensuring that subsequent analyses fully account for data uncertainty and potential bias.

10.Data visualization

| Name | Cite |
| --- | --- |
| bamp | Schmid V, Geressen F, Held L, Rainer E (2022). _ bamp: Bayesian Age-Period-Cohort Modeling and Prediction_. R package version 2.1.3, https://cran.r-project.org/web/packages/bamp/index.html. |
| data.table | Dowle M, Srinivasan A (2023). _data.table: Extension of `data.frame`_. R package version 1.14.8, <https://CRAN.R-project.org/package=data.table>. |
| digest | Lucas DEwcbA, Tuszynski J, Bengtsson H, Urbanek S, Frasca M, Lewis B, Stokely M, Muehleisen H, Murdoch D, Hester J, Wu W, Kou Q, Onkelinx T, Lang M, Simko V, Hornik K, Neal R, Bell K, de Queljoe M, Suruceanu I, Denney B, Schumacher D, Chang. aW (2022). _digest: Create Compact Hash Digests of R Objects_. R package version 0.6.31, <https://CRAN.R-project.org/package=digest>. |
| gglot2 | H. Wickham. ggplot2: Elegant Graphics for Data Analysis. Springer-Verlag New York, 2016. |
| dplyr | Wickham H, François R, Henry L, Müller K, Vaughan D (2023). _dplyr: A Grammar of Data Manipulation_. R package version 1.1.0, <https://CRAN.R-project.org/package=dplyr>. |
| forcats | Wickham H (2023). _forcats: Tools for Working with Categorical Variables (Factors)_. R package version 1.0.0, <https://CRAN.R-project.org/package=forcats>. |
| ggnewscale | Campitelli E (2022). _ggnewscale: Multiple Fill and Colour Scales in 'ggplot2'_. R package version 0.4.8, <https://CRAN.R-project.org/package=ggnewscale>. |
| ggrepel | Slowikowski K (2023). _ggrepel: Automatically Position Non-Overlapping Text Labels with 'ggplot2'_. R package version 0.9.3, <https://CRAN.R-project.org/package=ggrepel>. |
| ggsci | Xiao N (2023). _ggsci: Scientific Journal and Sci-Fi Themed Color Palettes for 'ggplot2'_. R package version 3.0.0, <https://CRAN.R-project.org/package=ggsci>. |
| inla | Krainski E T, Lindgren F, Rue H (2024). _INLAspacetime: Spatial and Spatio-Temporal Models using 'INLA'_. R package version 0.1.10, https://cran.r-project.org/web/packages/INLAspacetime/index.html |
| lubridate | Garrett Grolemund, Hadley Wickham (2011). _ Dates and Times Made Easy with lubridate. Journal of Statistical Software, 40(3), 1-25. URL <https://www.jstatsoft.org/v40/i03/>. |
| nordpred | Moller B, Weedon-Fekjaer H (2019) ._ nordpred: Fit power5 and poisson Age-Period-Cohort models to calculate prediction of cancer incidence and mortality_. R package version 1.1, URL http://www.kreftregisteret.no/software/nordpred/. |
| patchwork | Pedersen T (2022). _patchwork: The Composer of Plots_. R package version 1.1.2, <https://CRAN.R-project.org/package=patchwork>. |
| purrr | Wickham H, Henry L (2023). _purrr: Functional Programming Tools_. R package version 1.0.1, <https://CRAN.R-project.org/package=purrr>. |
| RColorBrewer | Neuwirth E (2022). _RColorBrewer: ColorBrewer Palettes_. R package version 1.1-3, <https://CRAN.R-project.org/package=RColorBrewer>. |
| readr | Wickham H, Hester J, Bryan J (2023). _readr: Read Rectangular Text Data_. R package version 2.1.4, <https://CRAN.R-project.org/package=readr>. |
| readxl | Wickham H, Bryan J (2023). _readxl: Read Excel Files_. R package version 1.4.2, <https://CRAN.R-project.org/package=readxl>. |
| sf | Pebesma, E., 2018. Simple Features for R: Standardized Support for Spatial Vector Data. The R Journal 10 (1), 439-446, <https://doi.org/10.32614/RJ-2018-009> Pebesma, E., & Bivand, R. (2023). Spatial Data Science: With Applications in R (1st ed.). Chapman and Hall/CRC. <https://doi.org/10.1201/9780429459016> |
| snow | Tierney L, Rossini AJ, Li N, Sevcikova H (2021). _snow: Simple Network of Workstations_. R package version 0.4-4, <https://CRAN.R-project.org/package=snow>. |
| snowfall | Knaus J (2022). _snowfall: Easier Cluster Computing (Based on 'snow')_. R package version 1.84-6.2, <https://CRAN.R-project.org/package=snowfall>. |
| stringr | Wickham H (2022). _stringr: Simple, Consistent Wrappers for Common String Operations_. R package version 1.5.0, <https://CRAN.R-project.org/package=stringr>. |
| tibble | Müller K, Wickham H (2022). _tibble: Simple Data Frames_. R package version 3.1.8, <https://CRAN.R-project.org/package=tibble>. |
| tidyr | Wickham H, Vaughan D, Girlich M (2023). _tidyr: Tidy Messy Data_. R package version 1.3.0, <https://CRAN.R-project.org/package=tidyr>. |
| tidyverse | Wickham H, Averick M, Bryan J, Chang W, McGowan LD, François R, Grolemund G, Hayes A, Henry L, Hester J, Kuhn M, Pedersen TL, Miller E, Bache SM, Müller K, Ooms J, Robinson D, Seidel DP, Spinu V, Takahashi K, Vaughan D, Wilke C, Woo K, Yutani H (2019). “Welcome to the tidyverse.” _Journal of Open Source Software_, *4*(43), 1686. doi:10.21105/joss.01686 <https://doi.org/10.21105/joss.01686>. |
| viridis | Simon Garnier, Noam Ross, Robert Rudis, Antônio P. Camargo, Marco Sciaini, and Cédric Scherer (2021). Rvision - Colorblind-Friendly Color Maps for R. R package version 0.6.2 |
| viridisLite | Simon Garnier, Noam Ross, Robert Rudis, Antônio P. Camargo, Marco Sciaini, and Cédric Scherer (2022). Rvision - Colorblind-Friendly Color Maps for R. R package version 0.4.1 |

**Table S1.** **SDI quintiles for countries estimated in GBD 2021**

| SDI quintile | Locations included based on SDI values in 2021 from GBD 2021 results |
| --- | --- |
| Low SDI（0.00-0.47） | Afghanistan, Benin, Burkina Faso, Burundi, Central African Republic, Chad, Cote d'lvoire, Democratic Republic of the Congo, Eritrea, Ethiopia, Gambia, Guinea, Guinea-Bissau, Haiti, Liberia, Madagascar, Malawi, Mali, Mozambique, Nepal, Niger, Pakistan, Papua New Guinea, Rwanda, Senegal, Sierra Leone, Solomon Islands, Somalia, South Sudan, Togo, Uganda, United Republic of Tanzania, Yemen |
| Low-middle SDI（0.47-0.62） | Angola, Bangladesh, Belize, Bhutan, Bolivia (Plurinational State of), Cabo Verde, Cambodia, Cameroon, Comoros, Congo, Democratic People's Republic of Korea, Djibouti, Dominican Republic, El Salvador, Eswatini, Ghana, Guatemala, Honduras, India, Kenya, Kiribati, Kyrgyzstan, Lao People's Democratic Republic, Lesotho, Maldives, Marshall Islands, Mauritania, Micronesia (Federated States of), Mongolia, Morocco, Myanmar, Nicaragua, Nigeria, Palestine, Sao Tome and Principe, Sudan, Tajikistan, Timor-Leste, Tuvalu, Vanuatu, Venezuela (Bolivarian Republic of), Zambia, Zimbabwe |
| Middle SDI（0.62-0.71） | Albania, Algeria, Armenia, Azerbaijan, Botswana, Brazil, China, Colombia, Costa Rica, Cuba, Ecuador, Egypt, Equatorial Guinea, Fiji, Gabon, Grenada, Guyana, Indonesia, Iran (Islamic Republic of), Iraq, Jamaica, Mexico, Namibia, Nauru, Panama, Paraguay, Peru, Philippines, Saint Lucia, Saint Vincent and the Grenadines, Samoa, South Africa, Suriname, Syrian Arab Republic, Thailand, Tokelau, Tonga, Tunisia, Turkmenistan, Uzbekistan, Viet Nam |
| High-middle SDI（0.71-0.81） | American Samoa, Antigua and Barbuda, Argentina, Bahamas, Bahrain, Barbados, Belarus, Bosnia and Herzegovina, Bulgaria, Chile, Cook Islands, Croatia, Dominica, Georgia, Greece, Greenland, Hungary, Israel, Italy, Jordan, Kazakhstan, Lebanon, Libya, Malaysia, Malta, Mauritius, Montenegro, Niue, North Macedonia, Northern Mariana Islands, Oman, Palau, Poland, Portugal, Republic of Moldova, Romania, Russian Federation, Saint Kitts and Nevis, Saudi Arabia, Serbia, Seychelles, Spain, Sri Lanka, Trinidad and Tobago, Turkey, Ukraine, United States Virgin Islands, Uruguay |
| High SDI（0.81-1.00） | Andorra, Australia, Austria, Belgium, Bermuda, Brunei Darussalam, Canada, Cyprus, Czechia, Denmark, Estonia, Finland, France, Germany, Guam, Iceland, Ireland, Japan, Kuwait, Latvia, Lithuania, Luxembourg, Monaco, Netherlands, New Zealand, Norway, Puerto Rico, Qatar, Republic of Korea, San Marino, Singapore, Slovakia, Slovenia, Sweden, Switzerland, Taiwan (Province of China), United Arab Emirates, United Kingdom, United States of America |

Abbreviation: SDI, socio-demographic index.

**Table S2. Global and regional death of NHL in middle-aged and older populations between 1990 and 2021.**

| **Location** | **Death cases** | | | **Age-standardized mortality rates** | | |
| --- | --- | --- | --- | --- | --- | --- |
|  | **1990** | **2021** | **Change Percent (%)** | **1990** | **2021** | **EAPC (95% CI)** |
| Global | 95493.73(89968.07,103284.14) | 204021.09(185987.81,219599.59) | 113.65 | 14.22(13.40,15.38) | 13.73(12.52,14.78) | -0.33(-0.45,-0.21) |
| **Gender** |  |  |  |  |  |  |
| Male | 52071.61(48984.03,58456.10) | 113981.06(104091.59,126448.28) | 118.89 | 16.72(15.73,18.77) | 16.29(14.88,18.08) | -0.22(-0.32,-0.12) |
| Female | 43422.11(39765.54,46341.16) | 90040.03(78730.55,97389.80) | 107.36 | 12.06(11.05,12.87) | 11.45(10.01,12.38) | -0.49(-0.64,-0.33) |
| **SDI** |  |  |  |  |  |  |
| High SDI | 45744.36(43004.27,47212.62) | 80252.04(70773.05,85684.06) | 75.44 | 24.53(23.06,25.32) | 23.26(20.51,24.84) | -0.56(-0.74,-0.38) |
| High-middle SDI | 19395.94(18238.86,21203.69) | 40910.55(37149.04,45121.67) | 110.92 | 11.24(10.57,12.29) | 11.80(10.72,13.02) | 0.10(-0.04,0.23) |
| Middle SDI | 16288.53(14907.01,19320.49) | 47000.88(42035.16,52694.16) | 188.55 | 9.38(8.59,11.13) | 10.00(8.95,11.21) | 0.09(0.01,0.17) |
| Low-middle SDI | 9106.22(7841.16,11415.66) | 25290.35(22526.33,31698.98) | 177.73 | 9.03(7.78,11.32) | 10.49(9.34,13.15) | 0.38(0.30,0.45) |
| Low SDI | 4860.72(3904.03,5755.48) | 10378.12(9007.68,12332.87) | 113.51 | 13.03(10.46,15.43) | 12.65(10.98,15.03) | -0.27(-0.37,-0.17) |
| **Regions** |  |  |  |  |  |  |
| Andean Latin America | 539.17(473.80,642.94) | 2147.19(1732.77,2638.93) | 298.24 | 16.07(14.12,19.16) | 21.67(17.49,26.64) | 1.00(0.82,1.17) |
| Australasia | 1213.84(1147.66,1277.64) | 2267.09(2000.08,2482.81) | 86.77 | 30.81(29.13,32.43) | 25.66(22.64,28.10) | -1.11(-1.31,-0.90) |
| Caribbean | 724.03(682.25,771.13) | 1300.71(1154.51,1451.68) | 79.65 | 16.80(15.83,17.89) | 14.05(12.47,15.68) | -0.47(-0.63,-0.32) |
| Central Asia | 410.57(378.82,448.12) | 666.40(578.23,751.79) | 62.31 | 5.13(4.74,5.60) | 4.58(3.97,5.17) | -0.39(-0.58,-0.21) |
| Central Europe | 2751.56(2641.86,2896.40) | 5600.77(5125.00,5994.75) | 103.55 | 10.38(9.96,10.92) | 15.13(13.84,16.19) | 1.10(0.84,1.37) |
| Central Latin America | 1365.38(1318.45,1399.91) | 5460.39(4866.54,6067.49) | 299.92 | 10.06(9.72,10.32) | 12.77(11.38,14.19) | 0.50(0.36,0.63) |
| Central Sub-Saharan Africa | 381.82(273.84,531.95) | 913.68(643.97,1329.46) | 139.30 | 10.15(7.28,14.15) | 10.13(7.14,14.73) | -0.12(-0.23,-0.01) |
| East Asia | 13819.65(11907.04,17326.80) | 34234.58(27346.59,41231.86) | 147.72 | 9.28(7.99,11.63) | 8.73(6.97,10.52) | -0.20(-0.36,-0.04) |
| Eastern Europe | 3409.63(3299.88,3561.99) | 6593.22(6080.84,7112.93) | 93.37 | 6.97(6.75,7.29) | 10.62(9.80,11.46) | 1.45(1.31,1.60) |
| Eastern Sub-Saharan Africa | 2516.47(2017.41,2975.65) | 5303.03(4484.81,6434.64) | 110.73 | 20.69(16.58,24.46) | 19.61(16.59,23.80) | -0.38(-0.47,-0.29) |
| High-income Asia Pacific | 5341.30(4990.83,5684.24) | 16946.32(14196.40,18583.48) | 217.27 | 15.27(14.27,16.26) | 24.04(20.14,26.36) | 1.43(1.33,1.53) |
| High-income North America | 20749.38(19254.28,21507.31) | 29213.36(25739.15,30977.49) | 40.79 | 35.82(33.24,37.13) | 25.96(22.87,27.53) | -1.69(-1.96,-1.42) |
| North Africa and Middle East | 3031.47(2489.77,3840.52) | 8618.97(7415.17,10494.72) | 184.32 | 10.73(8.81,13.59) | 11.31(9.73,13.77) | 0.20(0.11,0.29) |
| Oceania | 26.62(20.77,35.63) | 71.23(55.39,96.48) | 167.58 | 5.53(4.32,7.41) | 5.77(4.49,7.82) | 0.16(0.10,0.21) |
| South Asia | 8688.18(7244.51,10210.22) | 25813.92(22938.09,31106.64) | 197.12 | 9.15(7.63,10.75) | 10.40(9.24,12.53) | 0.21(0.10,0.33) |
| Southeast Asia | 4045.33(3526.00,5218.87) | 11768.20(10048.99,15974.89) | 190.91 | 9.55(8.33,12.33) | 10.27(8.77,13.95) | 0.01(-0.11,0.13) |
| Southern Latin America | 1397.05(1332.00,1459.00) | 2462.70(2233.45,2660.22) | 76.28 | 17.64(16.82,18.42) | 16.73(15.18,18.08) | -0.30(-0.73,0.13) |
| Southern Sub-Saharan Africa | 433.43(359.99,524.20) | 1385.45(1142.42,1543.54) | 219.65 | 9.80(8.14,11.85) | 14.23(11.73,15.86) | 1.27(0.86,1.69) |
| Tropical Latin America | 1656.23(1573.66,1711.93) | 5169.48(4737.31,5431.63) | 212.12 | 10.94(10.39,11.31) | 11.67(10.69,12.26) | -0.02(-0.31,0.27) |
| Western Europe | 21709.95(20516.78,22466.74) | 34874.36(30555.73,37490.95) | 60.64 | 22.36(21.13,23.13) | 23.38(20.49,25.14) | -0.11(-0.28,0.07) |
| Western Sub-Saharan Africa | 1282.67(1029.17,1504.58) | 3210.04(2462.77,3723.50) | 150.26 | 8.89(7.13,10.42) | 9.99(7.66,11.58) | 0.27(0.18,0.36) |

**Abbreviations**: EAPC, estimated annual percentage changes; SDI, Sociodemographic index.

EAPCs represent the annual percentage change and their 95% confidence intervals in age-standardized rates during 30 years from 1990 to 2021.

**Table S3. Global and regional DALYs of NHL in middle-aged and older populations between 1990 and 2021.**

| **Location** | **DALYs cases** | | | **Age-standardized DALYs rates** | | |
| --- | --- | --- | --- | --- | --- | --- |
|  | **1990** | **2021** | **Change Percent (%)** | **1990** | **2021** | **EAPC (95% CI)** |
| **Global** | 2109528.76(1990621.56,2298029.47) | 4248298.55(3922407.88,4597406.78) | 101.39 | 314.19(296.48,342.26) | 285.89(263.96,309.38) | -0.52(-0.63,-0.41) |
| **Gender** |  |  |  |  |  |  |
| Male | 1203370.41(1132007.62,1349200.59) | 2459901.19(2265888.86,2739042.35) | 104.42 | 386.34(363.42,433.15) | 351.66(323.92,391.56) | -0.43(-0.52,-0.34) |
| Female | 906158.36(836300.66,969204.26) | 1788397.35(1603066.83,1934623.96) | 97.36 | 251.75(232.34,269.27) | 227.40(203.83,245.99) | -0.66(-0.81,-0.52) |
| **SDI** |  |  |  |  |  |  |
| High SDI | 943206.26(901039.61,972533.14) | 1477836.69(1336293.33,1568490.91) | 56.68 | 505.84(483.23,521.57) | 428.34(387.32,454.62) | -0.93(-1.11,-0.74) |
| High-middle SDI | 444449.14(419636.04,489302.01) | 883843.92(796241.38,976392.43) | 98.86 | 257.61(243.23,283.61) | 254.95(229.68,281.64) | -0.10(-0.20,0.00) |
| Middle SDI | 386809.75(352103.85,459665.29) | 1069764.05(954382.41,1203950.92) | 176.56 | 222.87(202.87,264.84) | 227.68(203.12,256.24) | -0.04(-0.12,0.05) |
| Low-middle SDI | 215825.12(185772.41,269979.70) | 574022.58(511614.00,721088.80) | 165.97 | 214.11(184.30,267.83) | 238.10(212.21,299.10) | 0.22(0.14,0.29) |
| Low SDI | 117058.91(93982.58,139053.26) | 238845.64(206869.88,285210.25) | 104.04 | 313.76(251.91,372.72) | 291.07(252.10,347.57) | -0.45(-0.57,-0.34) |
| **Regions** |  |  |  |  |  |  |
| Andean Latin America | 12035.85(10545.85,14263.52) | 46786.28(37469.32,57644.98) | 288.72 | 358.65(314.25,425.03) | 472.28(378.23,581.89) | 0.85(0.69,1.00) |
| Australasia | 25300.09(24051.36,26554.33) | 42034.76(37620.20,45493.25) | 66.14 | 642.21(610.52,674.05) | 475.82(425.84,514.96) | -1.44(-1.64,-1.24) |
| Caribbean | 15995.38(15052.19,17222.53) | 28935.43(25528.25,32581.45) | 80.90 | 371.15(349.26,399.62) | 312.53(275.73,351.91) | -0.43(-0.59,-0.28) |
| Central Asia | 10245.51(9511.46,11028.78) | 16633.06(14346.69,18799.47) | 62.34 | 128.10(118.92,137.89) | 114.32(98.60,129.21) | -0.48(-0.67,-0.29) |
| Central Europe | 62627.02(60359.76,65886.14) | 116282.39(106499.91,124499.32) | 85.67 | 236.15(227.60,248.44) | 314.04(287.62,336.23) | 0.81(0.55,1.06) |
| Central Latin America | 30520.69(29648.22,31297.70) | 119068.14(106620.48,132910.45) | 290.12 | 224.91(218.48,230.64) | 278.42(249.31,310.78) | 0.40(0.28,0.52) |
| Central Sub-Saharan Africa | 9584.82(6906.12,13308.15) | 22817.45(16073.59,32873.37) | 138.06 | 254.90(183.66,353.91) | 252.87(178.13,364.31) | -0.12(-0.26,0.02) |
| East Asia | 333536.33(285495.31,419531.65) | 791285.19(630454.14,963279.62) | 137.24 | 223.92(191.67,281.65) | 201.80(160.78,245.66) | -0.28(-0.47,-0.09) |
| Eastern Europe | 83189.16(80434.89,87312.14) | 153028.86(141777.40,166441.81) | 83.95 | 170.15(164.51,178.58) | 246.51(228.38,268.11) | 1.18(1.03,1.33) |
| Eastern Sub-Saharan Africa | 60177.78(47916.55,71636.88) | 121507.39(101527.97,149148.60) | 101.91 | 494.65(393.87,588.85) | 449.40(375.51,551.63) | -0.54(-0.64,-0.45) |
| High-income Asia Pacific | 115791.40(109623.06,122884.95) | 282550.11(247914.12,306798.03) | 144.02 | 331.13(313.49,351.42) | 400.76(351.63,435.15) | 0.54(0.44,0.65) |
| High-income North America | 424756.59(400981.29,439404.79) | 556343.31(509198.01,588103.50) | 30.98 | 733.25(692.21,758.54) | 494.38(452.48,522.60) | -1.90(-2.15,-1.65) |
| North Africa and Middle East | 70075.53(57477.47,90077.39) | 193726.37(165687.17,236500.57) | 176.45 | 247.93(203.36,318.70) | 254.12(217.34,310.23) | 0.04(-0.02,0.11) |
| Oceania | 628.79(482.60,847.37) | 1653.15(1267.45,2253.57) | 162.91 | 130.70(100.31,176.14) | 133.95(102.70,182.60) | 0.14(0.10,0.19) |
| South Asia | 208239.58(174627.96,244750.95) | 581126.89(515650.39,702209.16) | 179.07 | 219.33(183.93,257.79) | 234.05(207.68,282.81) | -0.02(-0.16,0.11) |
| Southeast Asia | 96196.27(83013.02,124061.76) | 276263.96(235652.67,373528.88) | 187.19 | 227.19(196.06,293.00) | 241.16(205.71,326.07) | -0.00(-0.09,0.09) |
| Southern Latin America | 30904.78(29484.79,32292.72) | 50278.49(45874.72,54225.27) | 62.69 | 390.14(372.21,407.66) | 341.66(311.73,368.48) | -0.53(-0.91,-0.15) |
| Southern Sub-Saharan Africa | 9680.20(8034.57,11688.82) | 31976.01(26136.88,35860.01) | 230.32 | 218.77(181.58,264.17) | 328.45(268.48,368.35) | 1.42(1.00,1.84) |
| Tropical Latin America | 38142.53(36563.02,39326.18) | 110987.14(103213.23,116077.66) | 190.98 | 251.91(241.48,259.73) | 250.55(233.00,262.04) | -0.27(-0.55,0.00) |
| Western Europe | 442458.94(423863.08,456108.60) | 630974.40(562208.20,674078.32) | 42.61 | 455.62(436.47,469.67) | 423.09(376.98,451.99) | -0.52(-0.72,-0.33) |
| Western Sub-Saharan Africa | 29441.52(23664.95,34597.36) | 74039.77(56758.24,86689.14) | 151.48 | 203.95(163.94,239.67) | 230.34(176.58,269.70) | 0.28(0.19,0.36) |

**Abbreviations**: EAPC, estimated annual percentage changes; SDI, Sociodemographic index; DALYs: Disability-adjusted life-years.

EAPCs represent the annual percentage change and their 95% confidence intervals in age-standardized rates during 30 years from 1990 to 2021.
